# Light-driven liquid metal nanotransformers for biomedical theranostics

**DOI:** 10.1038/ncomms15432

**Published:** 2017-05-31

**Authors:** Svetlana A. Chechetka, Yue Yu, Xu Zhen, Manojit Pramanik, Kanyi Pu, Eijiro Miyako

**Affiliations:** 1 Department of Materials and Chemistry, Nanomaterials Research Institute (NMRI), National Institute of Advanced Industrial Science and Technology (AIST), Central 5, 1-1-1 Higashi, Tsukuba, Ibaraki 305-8565, Japan; 2 School of Chemical and Biomedical Engineering, Nanyang Technological University (NTU), Singapore 637457, Singapore

## Abstract

Room temperature liquid metals (LMs) represent a class of emerging multifunctional materials with attractive novel properties. Here, we show that photopolymerized LMs present a unique nanoscale capsule structure characterized by high water dispersibility and low toxicity. We also demonstrate that the LM nanocapsule generates heat and reactive oxygen species under biologically neutral near-infrared (NIR) laser irradiation. Concomitantly, NIR laser exposure induces a transformation in LM shape, destruction of the nanocapsules, contactless controlled release of the loaded drugs, optical manipulations of a microfluidic blood vessel model and spatiotemporal targeted marking for X-ray-enhanced imaging in biological organs and a living mouse. By exploiting the physicochemical properties of LMs, we achieve effective cancer cell elimination and control of intercellular calcium ion flux. In addition, LMs display a photoacoustic effect in living animals during NIR laser treatment, making this system a powerful tool for bioimaging.

## Abstract (web summary)

Liquid metals are excellent candidate materials for biomedicine, owing to their intriguing optical properties and chemical stability. Here, the authors design multifunctional theranostic liquid metal nanocapsules that, upon irradiation, generate heat and reactive oxygen species and change shape to release drugs.

Owing to their desirable physical properties, such as high conductivity or favourable flexibility, room temperature liquid metals (LMs) have been shown to possess unique advantages in a variety of research fields^1^,^2^. Mercury is a well-known room temperature LM; however, its elevated toxicity is a major impediment to widespread application. LMs such as gallium, gallium-indium eutectic alloys (EGaIn: 75% Ga, 25% In (wt wt^−1^)), and gallium-indium-tin alloys (Galinstan: 68.5% Ga, 21.5% In and 10% Sn (wt wt^−1^)), with melting points of 29.8, 15.7 and 10.7 °C, respectively, have received increasing attention because they are generally chemically stable and do not react with water at room temperature. In addition, previous studies have shown that such alloys are safe for humans under normal circumstances^3^,^4^,^5^,^6^,^7^,^8^,^9^,^10^. Currently, there is a significant interest towards the chemical^3^,^4^,^5^ and morphological characterization of these emerging LMs^5^,^6^,^7^,^8^,^9^. Furthermore, as opposed to mercury and solid metals such as gold or silver nanoparticles whose optical properties have been extensively investigated, those of promising LMs have not been sufficiently explored^11^,^12^,^13^,^14^. Current major challenges are the simple production of LM spherical droplets and chemical functionalization of LM nanoconjugates^4^,^10^,^15^. They require not only exquisite synthesis strategies but also unravelling the mystery of the unique properties in LMs. Many research groups have attempted to improve this synthesis and chemical functionalization of LMs using diverse procedures such as microfluidic productions^15^ and chemical modification with functional molecules^4^,^10^. Among these approaches, we believe that chemical modification is an excellent candidate for modulating the optical properties of LMs, especially for large-scale production. The next logical step is to test these new chemical functionalized LMs in a variety of biological applications^10^. Indeed, there is currently great interest in understanding and achieving biomedical approaches of LMs in physiological environments, related to health recovery, imaging or biocompatibility animal evaluation of LMs^10^,^16^,^17^,^18^. Analyses of the biocompatibility, multifunctionalization with bioactive molecules and spatiotemporal control of activities of the chemically modified LM nanocomplexes will be also a milestone for integrating these new materials with numerous biological applications.

In this study, we demonstrate that EGaIn photopolymerized with functional phospholipids shows a unique nanocapsule structure, characterized by high water dispersibility and low toxicity. Chemically functionalized EGaIn nanocapsules absorb NIR light to generate thermal energy and reactive oxygen species (ROS). NIR irradiation causes changes in LM shape and the destruction of nanocapsules for the controlled release of drug molecules, optical manipulations of a microfluidic blood vessel model and targeted marking for X-ray-enhanced imaging in organs and a living mouse. LM nanocomplexes can be applied to cancer treatment, and can also control the spatiotemporal stimulation of ion channels in targeted cells. The photoacoustic (PA) effect of photo-induced LMs can be used for bioimaging. We also analyse the photo-triggered LM thermodynamics (morphological changes) to show that it is consistent with the proposed LM nanocapsules disintegration and transformation mechanism. The current work clearly shows that the LM nanocapsules simultaneously display the above-mentioned multiple physicochemical performances with good water dispersibility, superior photothermal conversion efficiency (52%), high biocompatibility, excellent photothermal stability, fine thermal and freezing resistances, a wide range of light energy absorbance for photothermal conversion and powerful PA performance.

## Results

### Synthesis and structural characterization of LM nanocapsules

**Figure 1:**
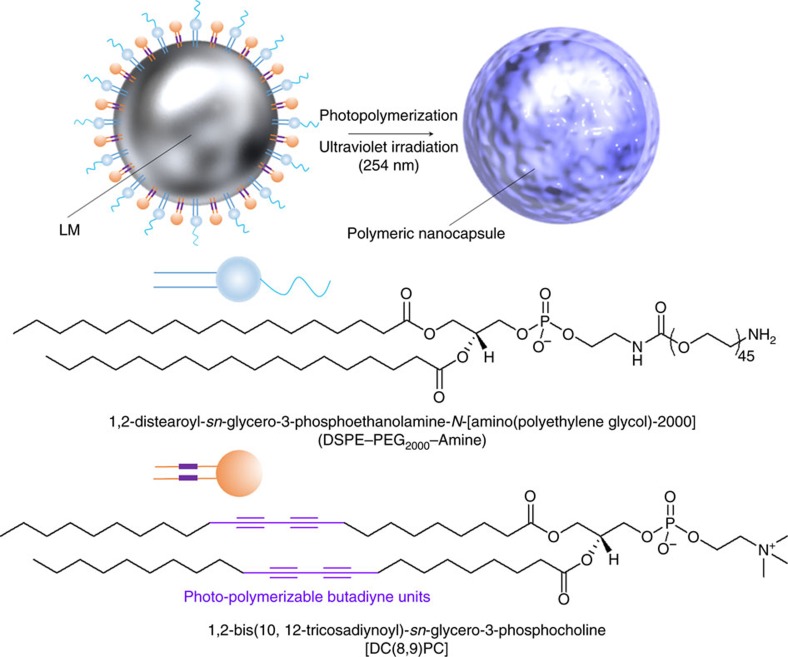
Schematic illustration of a transformable LM nanocapsule. Components of LM nanocapsule are EGaIn, DSPE-PEG_2000_-Amine and DC(8,9)PC. Polymeric core-shell structure encapsulating LM is prepared by crosslinking of butadiyne moieties from DC(8,9)PC with 254 nm ultraviolet irradiation.

**Figure 2:**
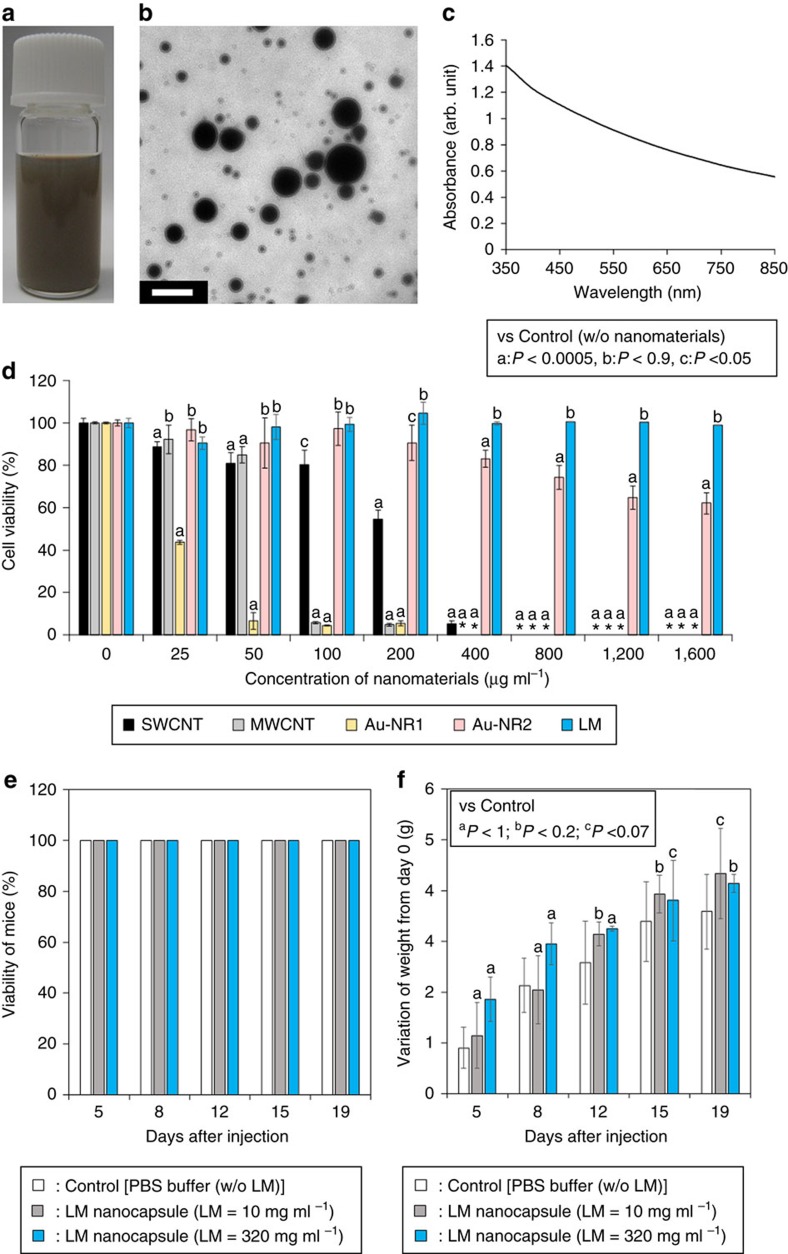
Characterization of LM nanocapsules. (**a**) Vial containing an aqueous suspension of LM nanocapsules (LM concentration: 115 μg ml^−1^). (**b**) TEM image of LM nanocapsules. Scale bar, 200 nm. (**c**) Ultraviolet–visible–NIR absorbance spectrum of LM nanocapsules (LM concentration: 500 μg ml^−1^). (**d**) Cell viability following treatment with various nanomaterials. SWCNT, single-walled carbon nanotube; MWCNT, multi-walled carbon nanotube; Au-NR1, methyl-terminated-hydrophilic polymer-conjugated gold nanorod; Au-NR2, methoxy-PEG_2000_-SH modified gold nanorod. Error bars represent s.d.'s of five separate measurements. *N.D.: not determined because all of cells were dead. The standardized percentage of mitochondrial enzyme activity compared with control. Statistical analyses were performed using ANOVA (Tukey's test). ^a^*P*<0.0005; ^b^*P*<0.9; ^c^*P*<0.05. (**e**) Viability and (**f**) body weight of the mice (*N*=5) following the injection of LM nanocapsules observed for 19 days. Data represent the mean of five determinations; error bars show the s.d. ^a^*P*<1; ^b^*P*<0.2; ^c^*P*<0.07.

A schematic illustration of the synthesis of a representative LM nanocapsule is shown in Fig. 1. Unfortunately, LM itself is not soluble in water. Instead, thiol-terminated surfactants, such as 1-dodecanethiol^4^ and sugar-based thiolates^10^, have been typically used to obtain LM suspensions; both however, present drawbacks. 1-dodecanethiol is water-insoluble and therefore poorly biocompatible. The synthesis of sugar-based thiolates requires multiple steps and uses costly materials. Moreover, thiol compounds are difficult to handle because they are easily oxidized and inactivate metalloenzymes in living organisms, causing side effects and toxicity^19^. Therefore, development of a simple and convenient method for preparing water-dispersible LM nanoconjugates with high biocompatibility would be highly desirable for biomedical applications. To prepare a typical LM suspension, we mixed EGaIn with 1,2-distearoyl-*sn*-glycero-3-phosphoethanolamine-*N*-[amino(polyethylene glycol)-2,000] (DSPE-PEG_2000_-Amine) and 1,2-bis(10,12-tricosadiynoyl)-*sn*-glycero-3-phosphocholine (DC(8,9)PC) in water using pulse sonication for 10 min. Long alkyl chains, containing conjugated butadiyne (–C≡C–C≡C–) from DC(8,9)PC, self-assembled by crosslinking following photo-induced photochemical polymerization^20^,^21^,^22^. The formation of a spherical assembly of DSPE-PEG_2000_-Amine and DC(8,9)PC involved hydrophobic and π–π stacking interactions from aliphatic hydrocarbon chains and diyne groups. We expected that the polyethylene glycol (PEG) moieties and hydrogen bonds between cationic trimethyl amino groups and water molecules would enhance the solubility of LM in an aqueous solvent. We tested the dispersibility of DSPE-PEG_2000_-Amine and DC(8,9)PC functionalized LM after ultraviolet irradiation (DSPE-PEG_2000_-Amine-DC(8,9)PC-LM) and obtained a homogeneous solution (Fig. 2a). Synthesized DSPE-PEG_2000_-Amine-DC(8,9)PC-LM displayed high water dispersibility and no precipitations. The results of dynamic light scattering (DLS) showed that the hydrodynamic diameter of these LM nanoconjugates was ∼150 nm and size did not change for at least 3 days at 20 °C (Supplementary Fig. 1). Although some of the LM nanoconjugates formed precipitations at the bottom of a vial after incubation overnight, they are easily redispersed in water by simple vial shaking or vortex treatment and could be used for multiple cycles. Indeed, resuspended LM nanoconjugates displayed almost same DLS histogram as a freshly prepared sample without incubation (Supplementary Fig. 1b). High-resolution transmission electron microscopy (TEM) demonstrated the formation of spherical capsule-like structures (Fig. 2b). TEM exhibited a polydispersion of particle size of LM nanocapsule. The average diameter of dried LM nanocapsules (*N*=100) on a grid was about 90 nm from TEM observation (Supplementary Fig. 2). In addition, we found a thin ∼20 nm layer surrounding the LM nanospheres (Supplementary Fig. 3). We believe that this could act as a shell preventing reversible aggregation and size changes of LM nanocapsules. Presence of Ga and In elements in the nanocapsules were also confirmed by scanning TEM/energy dispersive X-ray spectrometer (EDS) (STEM/EDS) mapping (Supplementary Fig. 4). The ultraviolet–visible–NIR absorption spectrum of LM nanocapsules, from 350 to 800 nm, did not exhibit any peaks. This behaviour is quite similar to the previously reported LM colloidal solutions^4^ (Fig. 2c). Meanwhile, LM nanoconjugates prepared only with DSPE-PEG_2000_-Amine were seen to form numerous aggregates after a few days (Supplementary Fig. 5a). Ultraviolet absorbance measurements confirmed a lower LM concentration in solution of DSPE-PEG_2000_-Amine conjugates than of DSPE-PEG_2000_-Amine-DC(8,9)PC-LMs (Supplementary Fig. 5b). In addition, ultraviolet absorbance of DSPE-PEG_2000_-Amine and DC(8,9)PC modified LM nanoconjugate without ultraviolet irradiation also lower LM concentration rather than that of ultraviolet-irradiated DSPE-PEG_2000_-Amine-DC(8,9)PC-LMs (Supplementary Fig. 5b). Moreover, TEM images indicated that LM nanoconjugates without DC(8,9)PC lacked a uniform shell structure on their metallic surface (Supplementary Fig. 3f). This implied that they could easily fuse with each other to form large aggregates, as confirmed also by TEM (Supplementary Fig. 3). These results clearly indicated that photopolymerization of DC(8,9)PC could confer LMs a unique spherical nanostructure with high water dispersibility.

### Cytotoxicity of LM nanocapsules

Unlike mercury, EGaIn displays low toxicity and it has thus attracted considerable attention. Nevertheless, concerns about the toxicity of emerging nanomaterials remain^23^. We tested the possible toxicity of our preparation of DSPE-PEG_2000_-Amine-DC(8,9)PC-LM nanocapsules and compared it to other representative nanomaterials, such as single-walled carbon nanotubes (SWCNTs), multi-walled CNTs (MWCNTs) and gold nanorods (Au-NRs). Human cervical cancer (HeLa) cells were pre-incubated with these nanomaterials at different concentrations (25, 50, 100, 200, 400, 800, 1,200 and 1,600 μg ml^−1^) for 24 h. Before the experiments, SWCNTs and MWCNTs were functionalized with DSPE-PEG_2000_-Amine to obtain water-dispersible CNTs (DSPE-PEG_2000_-Amine-SWCNT and DSPE-PEG_2000_-Amine-MWCNT, respectively). Commercially available water-soluble Au-NRs (methyl-terminated-hydrophilic polymer conjugated Au-NR (Au-NR1) and methoxy-PEG_2000_-SH modified Au-NR (Au-NR2)) were used without functionalization. LM nanocapsules displayed little toxicity at high concentrations (>90% cells were alive) compared to other nanomaterials (Fig. 2d). In addition, high concentrated LM nanocapsules (up to 320 mg ml^−1^) injections did not affect the viability and body weight of mice up to 19 days (Fig. 2e,f). The low LM toxicity was also demonstrated by many researchers in both *in vitro*^10^ and *in vivo*^10^,^17^,^18^ studies. Given such high biocompatibility, we believe that LM nanocapsules would be appropriate for biomedical applications.

### Photothermal conversion of LM nanocapsules

**Figure 3:**
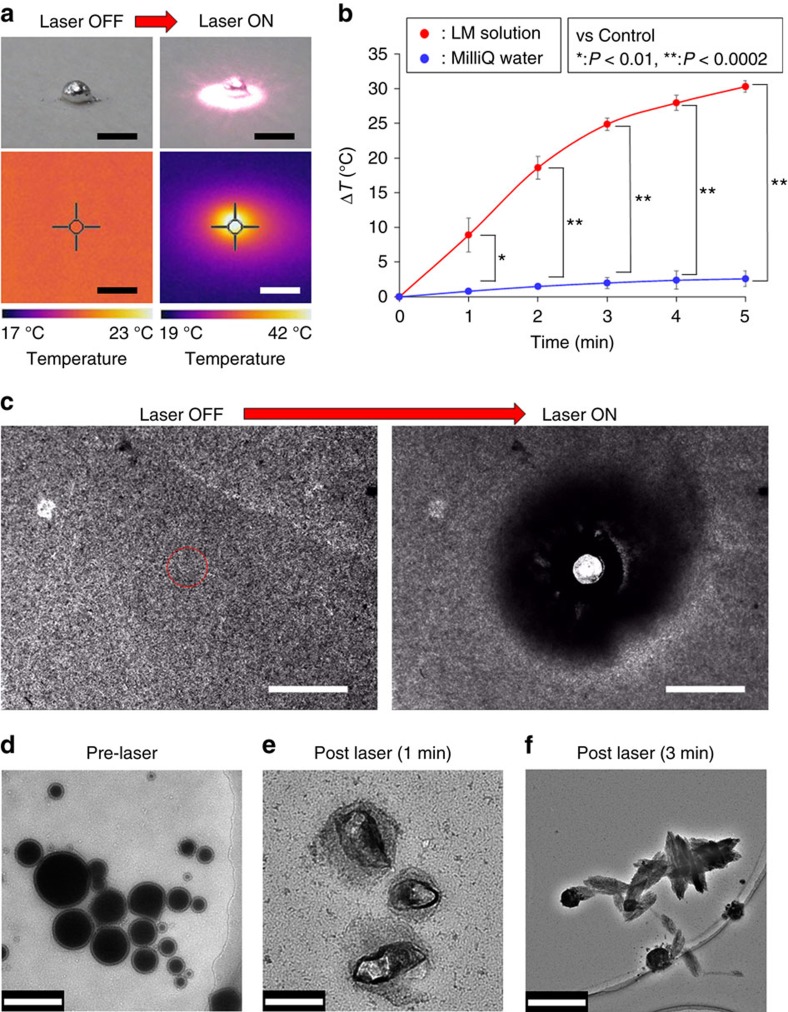
Laser-induced morphological changes in LM nanocapsules. (**a**) Visible light and thermographic images of a representative LM droplet (1 mg) before and after irradiation with a 785 nm NIR laser at 1 W (∼80 mW mm^−2^) for 5 min. Scale bars, 2 mm. (**b**) Thermal response of a 100 μg ml^−1^ DSPE-PEG_2000_-Amine-DC(8,9)PC-LM solution by a laser source (785 nm, 1 W, ∼80 mW mm^−2^). Error bars represent s.d.'s of three separate measurements. *P<0.01; **P<0.0002. (**c**) Laser-promoted degradation of DSPE-PEG_2000_-Amine-DC(8,9)PC-LM nanoconjugates before and after laser irradiation (808 nm, 564 mW, ∼287 μW μm^−2^). The red circle shows laser irradiation position and area. Scale bars, 100 μm. (**d**–**f**) TEM images of LM nanocapsules (**d**) before and after laser irradiation for (**e**) 1 and (**f**) 3 min (785 nm, 1 W, ∼80 mW mm^−2^). Scale bars, 200 nm (**d**); 100 nm (**e**); and 500 nm (**f**).

We had previously developed functional nanomaterials for biological applications using NIR light (650–1,500 nm) (refs 24, 25, 26, 27, 28). NIR technology is very attractive to the biomedical field because, except for water (absorbance wavelength, 1,480 nm), biological systems are relatively transparent to NIR light^29^. The photothermal properties of LMs treated with NIR light have received little attention, as studies have focused instead on physicochemical characteristics (for example, electrical properties) of interest for bioengineering purposes^30^,^31^,^32^,^33^. Here, we applied NIR laser light to explore the optical properties of LM. Figure 3a shows thermographic images of LM droplets (LM weight: 1 mg) before and after 1 W (80 mW mm^−2^) irradiation with a 785 nm NIR laser. Interestingly, after a 5 s irradiation time, the temperature of the LM droplet surface rose immediately to ∼42 °C; after 5 min of exposure the temperature was still ∼43 °C. The difference in temperature compared to time 0 (Δ*T*) was ∼23 °C (Supplementary Fig. 6). Importantly, the LM exhibited superior photothermal stability than a representative organic dye molecule (indocyanine green: ICG) that are normally used in NIR laser-promoted photodynamic therapy^34^,^35^. Indeed, any degradation in optical absorbance of LM against laser irradiation was not observed over a monitoring period of 1 h laser exposure in air (Supplementary Fig. 7). This clearly illustrated the robustness of the metal against photobleaching. Next, we looked at the photothermal properties of LM nanocapsules in solution during laser irradiation (Fig. 3b). Nanocapsules released a significantly larger amount of heat than water (used as a control). Interestingly, Δ*T* reached ≈30 °C after just 5 min of laser irradiation. It should be noted, that the weight of the nanocapsules (30 μg) was lower than that of the laser-heated droplet (1 mg) mentioned above. This is likely due to LM nanocapsules in solution presenting a combined larger surface area for light absorption than that of a single massive LM droplet. The photothermal conversion efficiency of the LM nanocapsules and commercial Au-NR1 were 52% and 17%, respectively (Supplementary Fig. 8 and see Methods for more details). The LM nanocapsules have also high heating and freezing stabilities probably due to the high flexibility and excellent robustness of LMs and soft polymer shell (Supplementary Fig. 9)^1^,^2^,^3^,^4^,^5^,^6^,^7^,^8^,^9^,^10^. These results clearly demonstrate that NIR laser-induced LM nanocapsules are able of converting light into high thermal energy with high photothermal conversion efficiency and superior heating and freezing resistances. The mechanism underlying NIR laser-triggered exothermy of LMs probably involves optically stimulated resonance that causes rapid heating, akin to that found in photo-induced metal nanoparticles and CNTs^36^,^37^.

### Light-induced transformation of LM nanocapsules

**Figure 4:**
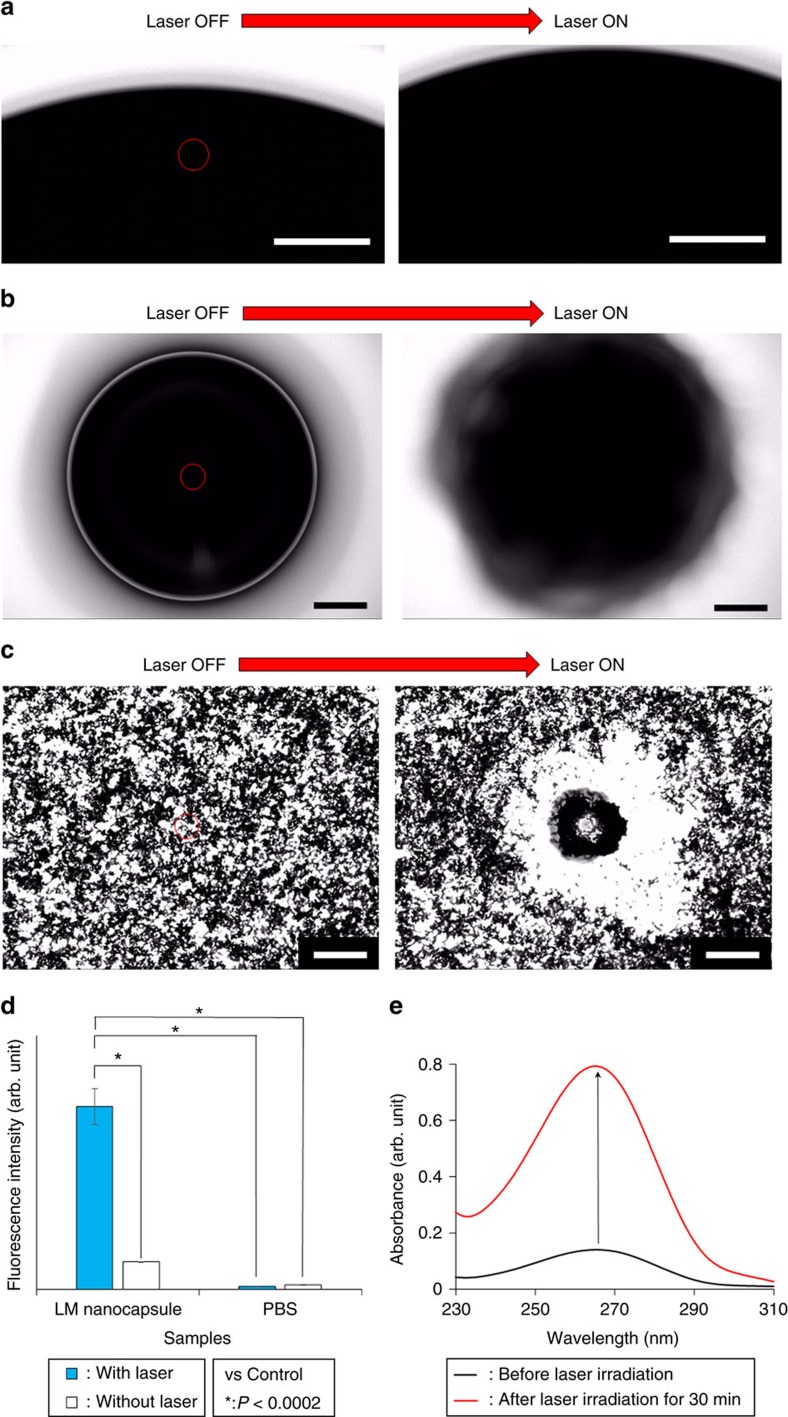
Optical and drug control releasing properties of laser-induced LM. Thermal expansion of laser-driven LM droplet in **a** the air (Scale bars, 150 μm) and (**b**) 0.1 M NaOH solution (Scale bars, 100 μm). (**c**) Morphological changes of LM aqueous suspension before and after laser irradiation. The red circles show laser irradiation position and area. Wavelength of laser=808 nm, laser power=564 mW (∼287 μW μm^−2^). Samples were prepared without DSPE-PEG_2000_-Amine and DC(8,9)PC. Scale bars, 100 μm. (**d**) ROS generation by photo-induced DSPE-PEG_2000_-Amine-DC(8,9)PC-LM after 30 min laser irradiation (785 nm, 1 W, ∼80 mW mm^−2^). Error bars represent s.d.'s of three separate measurements. *P<0.0002. (**e**) Controlled release of carmofur from laser-stimulated LM nanocapsules.

Surprisingly, the morphological changing (degradation) and thermal Brownian motion acceleration of LM nanocapsules were clearly observed via the optical microscope after laser irradiation (Fig. 3c and Supplementary Movie 1). A clear pop (indicating PA properties) was also heard from the laser-induced LM nanocapsules. TEM revealed that LM nanocapsules partially or completely collapsed after 1 and 3 min of laser exposure (Fig. 3d–f and Supplementary Fig. 10). We also confirmed the diameter changing of laser-induced LM nanocapsules by DLS measurements (Supplementary Fig. 11). DLS diagram of LM nanocapsules was shifted to larger particle size after laser irradiation due to the transformation of LMs. In addition, laser-induced LM droplet in the air was dynamically expanded on a slide glass probably because of the thermal expansion of LMs^38^,^39^,^40^ triggered by powerful photothermal property of them (Fig. 4a and Supplementary Movie 2). A spherical LM droplet in 0.1 M NaOH solution slightly moved on a slide glass and dramatically transformed amorphous structure after laser irradiation by thermal acceleration of Brownian motion and the above-mentioned thermal expansion of LMs (Fig. 4b and Supplementary Movie 3). Furthermore, pulse-sonicated LM aqueous suspension without DSPE-PEG_2000_-Amine and DC(8,9)PC immediately fused each other and transformed to massive accumulations during laser irradiation (Fig. 4c and Supplementary Movie 4). In fact, temperature of a Galinstan thermometer that is driven by thermal expansion mechanism was quickly increased by laser irradiation (Supplementary Fig. 12a). In addition, thermal expansion and shape changing behaviours of an EGaIn LM droplet could be also happened in accordance with the gravity within several seconds just after heating in air. Saturated final deformation of the heated-LM droplet required time at least for 40 s and high thermal energy ∼200 °C (Supplementary Fig. 12b,c and see Supplementary Movie 5). The surface temperature of a LM droplet on a glass slide was achieved to 254 °C after heating for 1 min (Supplementary Fig. 12d). Shape of the control LM droplet without heating was not changed at all. However, the shape changing phenomenon of this heated-LM droplet was relatively static rather than the laser-induced thermometer because a LM thermometer is basically equipped with optimal structures, such as capillary tube, fluid reservoir and anti-adhesion coating against LM, to cause dramatic movement of LM^38^. Interestingly, aqueous suspension of LM nanocapsules in a glass test tube was meanwhile rapidly formed precipitations after heating at lower temperature (130 °C). DLS showed that the heated-LM nanocapsules obviously increased particle size although Ultraviolet-crosslinked polymer nanocapsules without LMs did not exhibit any changing of their hydrodynamic diameter after heating (Supplementary Fig. 13a,b). Drastic structural destruction of the heated-LM nanocapsules was also confirmed by TEM (Supplementary Fig. 13c). Therefore, we believe that the heat-induced dynamic shape changing of the LM nanocapsule was principally derived from thermal expansion of LMs and small heat capacity of nanoscale objects.

### ROS generation from laser-induced LM nanocapsules

Lovell *et al*. have recently demonstrated that ROS generated by NIR laser-stimulated porphyrin-phospholipid could destroy liposomes by lipid oxidation^41^. Therefore, we hypothesize that laser-irradiated LM nanocapsules can produce ROS and destroy the lipid nanostructure as well. ROS generation was confirmed in horseradish peroxidase (HRP) -mediated reactions with the help of 2′,7′-dichlorodihydrofluorescein diacetate (H2DCFDA), a ROS marker (Fig. 4d). LM nanocapsules produced ROS during the laser treatment due to energy and electron transfer (types I and II reactions, respectively), as had been reported previously for Au-NRs and CNTs^42^,^43^. Control samples without LM nanocapsules did not show significant ROS generation. Hydrogen peroxide (H_2_O_2_) is well known as a potent oxidant and one of the type of non-radical ROS^44^. Thus, we focused our attention on the potential transformation behaviour of LM nanocapsules after H_2_O_2_ treatment by a Ultraviolet–visible–NIR spectrometer (Supplementary Fig. 14). The LM nanocapsule was able to keep high turbidity for the whole of the experimental period because it has very high water dispersibility for 3 days as also mentioned above, whereas the H_2_O_2_-treated LM nanocapsule was gradually precipitated over time. After all, the suspension of H_2_O_2_-treated LM nanocapsule was completely separated into grey precipitations and a clear solution after 24 h. These precipitations could not be redispersed in suspension at all by a vortex or vigorous shaking. Herein, we consider that the transformed precipitations were originally from LMs because of their high chemical stability and low water dispersibility. On the other hand, by incubating LM nanocapsules with a common enzyme, HRP and H_2_O_2_ under static conditions, these LM nanocapsules were effectively oxidized. Indeed, the transmittance of LM nanocapsules was rapidly increased and formed precipitations at the bottom of a vial after treatment with a combination of HRP and H_2_O_2_ for only 1 h. The formation of a highly oxidizing intermediate from this enzyme, known as Compound I, facilitates this biodegradation process^45^,^46^,^47^. The obtained data clearly indicate that ROS has capability to destruct the polymer shells on LMs although it takes time to completely decompose them because of strong crosslinked shell structures. In particular, oxidative enzymatic reaction with H_2_O_2_ potentially improves biodegradability of LM nanocapsules even though we need further investigations. Anyway, these results mark the first promising possibilities for LM nanocapsules to be degraded in environmentally relevant settings.

### Mechanism of light-driven LM transformation

Together, our data suggest that the mechanism underlying laser-triggered nanocapsule disintegration mainly derives from thermal expansion of LMs although ROS-induced partial lipid oxidation is little involved as a side reaction (Supplementary Fig. 15). Anyway, we believe that these dynamic transformations of laser-induced LMs have effective capacity to destruct the morphology of LM nanocapsules. At least, laser-induced nanocapsule disintegration and transformation may be exploited for the contactless remote-controlled transformation of LM and replace traditional contact-dependent electrical stimulation^6^ or the passive oxidation-reduction treatment of cells^10^. In addition, this light-promoted disintegration and transformation of LMs would be useful for laser welding technology^48^, targeted connecting for the transected nerves^16^,^31^ and electronics^49^ and non-contact wiring on electrodes of flexible diagnostic devices^50^ by a highly focused continuous wave (CW) laser beam with low power. We also consider that rapid morphological changings of laser-driven LM nanocapsules is promising for active drug delivery system in comparison to the slow and passive transformable properties^4^,^10^ of previous reported LM nanocomplexes under an acidic condition. We therefore expected the destruction of nanocapsules by NIR laser light to be useful for the controlled release of drugs at targeted locations. To test this hypothesis, we encapsulated carmofur, a pyrimidine analogue used as an antineoplastic agent^51^, into LM nanocapsules. Ultraviolet–visible–NIR spectra showed a characteristic peak from carmofur at ∼266 nm after encapsulation; this corresponded to a slight shift to the red compared to the original carmofur solution (peak at 259 nm), caused by interaction of the drug with the LM and lipid shell (Supplementary Fig. 16). A total of 38 μg ml^−1^ carmofur was released after NIR laser irradiation for 30 min (Fig. 4e). These data suggest that drug-loaded functionalized LM nanocapsules may be effective for targeted drug release during antitumour therapy or the regulation of cell metabolism.

### Laser-triggered LM transformation in a blood vessel model

**Figure 5:**
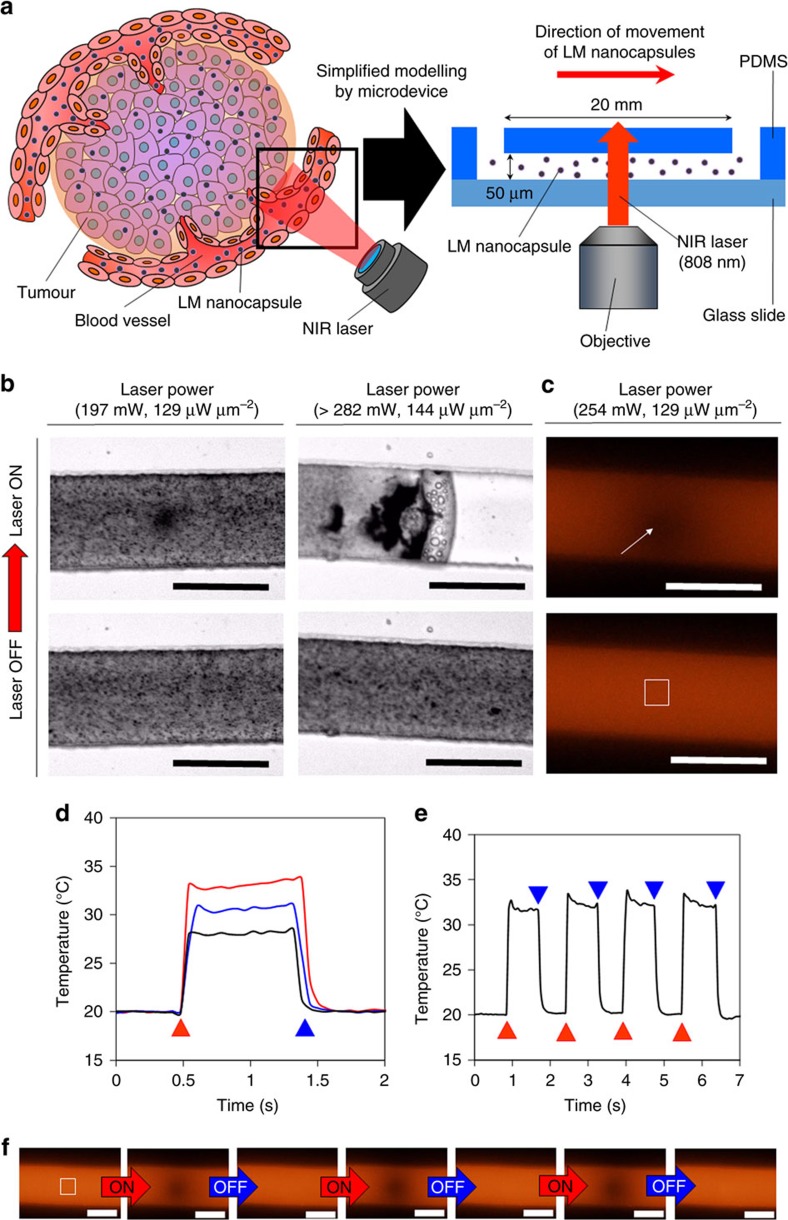
Optical control of transformation of LM nanocapsules in a microfluidic device. (**a**) Schematic illustration of the experimental system and design drawing of the microdevice based on a straight microchannel. Red arrows show the direction of LM nanocapsule movement. (**b**) Control of fluid mechanics by laser-induced transformation of LM nanocapsules in microdevice. Scale bars, 100 μm. (**c**) A direct observation of ultrafast temperature change in the microchannel. The white square indicates the location at which the temperature was analysed in **d**. The white arrow indicates the fluorescence quenching of rhodamine B by the photothermal property of LM nanocapsules. Magnification: × 20. Laser power: 254 mW (129 μW μm^−2^). Scale bars, 100 μm. (**d**) Temperature curves of the photo-induced LM nanocapsules under continuous NIR laser irradiation (wavelength: 808 nm) at various laser powers of 197 (black line), 226 (blue line) and 254 mW (red line) (100, 115 and 129 μW μm^−2^, respectively). (**e**) Highly precise thermal cycle by continuous laser ON–OFF switching. The red triangles and blue inverted triangles indicate the laser ‘ON' and ‘OFF' modes, respectively. (**f**) A direct observation of ultrafast microthermal control. Magnification: × 20. Laser power: 226 mW (115 μW μm^−2^). Wavelength: 808 nm. The white square indicates the location at which the temperature was analysed in **f**. Scale bars, 100 μm.

We further explored potential capabilities of the unique light-induced transformation mechanism of LM nanocapsules for biomedical theranostic applications. Blood vessels are involved in many biological processes, such as the transport of biomolecules, the maintenance of homoeostasis and the development of cancer metastases^52^. In particular, it is well known that control of flowing and temperature of blood vessels strictly effects on various human activities^52^. In addition, effective angiogenesis and blood flow inhibitors, that are key agents in the promotion of cancer, are urgently required to improve outcome in the cancer treatments^53^. Succeeding in the non-contact manipulation of flowing speed of substrates and temperature in blood vessels using light-induced transformation of LM nanocapsules would represent an innovative toolbox as a nano-microsurgical technology for solving the secrets of blood vessel biology, and it could be additionally exploited for the development of effective inhibiter for cancer angiogenesis and blood flowing. Therefore, we observed the transformation behaviour of laser-induced LM nanocapsules in the microfluidic devices as a model of blood vessels (Fig. 5). Microfluidic devices are typically used as a simple model for complicated blood vessel networks^54^,^55^. First, we prepared a polydimethylsiloxane (PDMS) microfluidic device based on a straight microchannel (width: 100 mm, depth: 50 mm) to investigate the microscale behaviour of LM nanocapsules by laser irradiation (Fig. 5a). LM nanocapsules were injected from an inlet using compressed air with a syringe, and nanocapsules were thereby quickly moving in the device by air pressure. Flowing speed of LM nanocapsules was then estimated about 571 μm s^−1^. When the moving LM nanocapsules in the microchannel was irradiated by an NIR laser (wavelength: 808 nm, laser power: 197 mW (∼100 μW μm^−2^), laser spot diameter: ∼50 μm), we immediately observed the transformation of LM nanocapsules caused by laser irradiation (Fig. 5b and Supplementary Movie 6). High-powered laser irradiation (>282 mW (∼144 μW μm^−2^)) induced massive LM aggregations and bubbles that immediately stopped the flowing of movement of LM nanocapsules. Upon laser NIR irradiation, we measured the temperature of a microspace utilizing rhodamine B molecules (Fig. 5c and Supplementary Movie 7)^24^. The fluorescence quenching of rhodamine B in the microchannel was observed almost immediately (below 0.03 s) upon NIR laser irradiation. In addition, the temperatures at laser powers of 197, 226 and 254 mW (100, 115 and 129 μW μm^−2^, respectively) increased from 20 to 28 °C and 34 and 38 °C, respectively (Fig. 5d). The thermal cycle in the microchannel was investigated by the sequential ‘ON' and ‘OFF' switching of the NIR laser at 226 mW (115 μW μm^−2^) (Fig. 5e,f and Supplementary Movie 7). We observed ultrafast and highly precise quenching and restoration of the fluorescence of rhodamine B in a microspace. The temperature change is reversible and can be cycled between the high- and low-temperature states at high speeds. Furthermore, thermal strain of microchannel was also observed at the centre position of highly focused-laser beam by powerful photothermal property of LM nanocapsules. Although this proof of concept utilized simple microfluidic blood vessel modelling, we believe that these laser-driven LM transformation behaviours would be effective for controlling temperature, flowing speed of substrates in real blood vessels, vasoconstrictor actions and inhibition for tumour angiogenesis and blood flowing, that will improve the therapeutic effects. Anyway, this unique property of the LM nanocapsules could be represent a completely unprecedented approach rather than previous works using general photothermal nanomaterials.

### Enchantment of X-ray contrast

**Figure 6:**
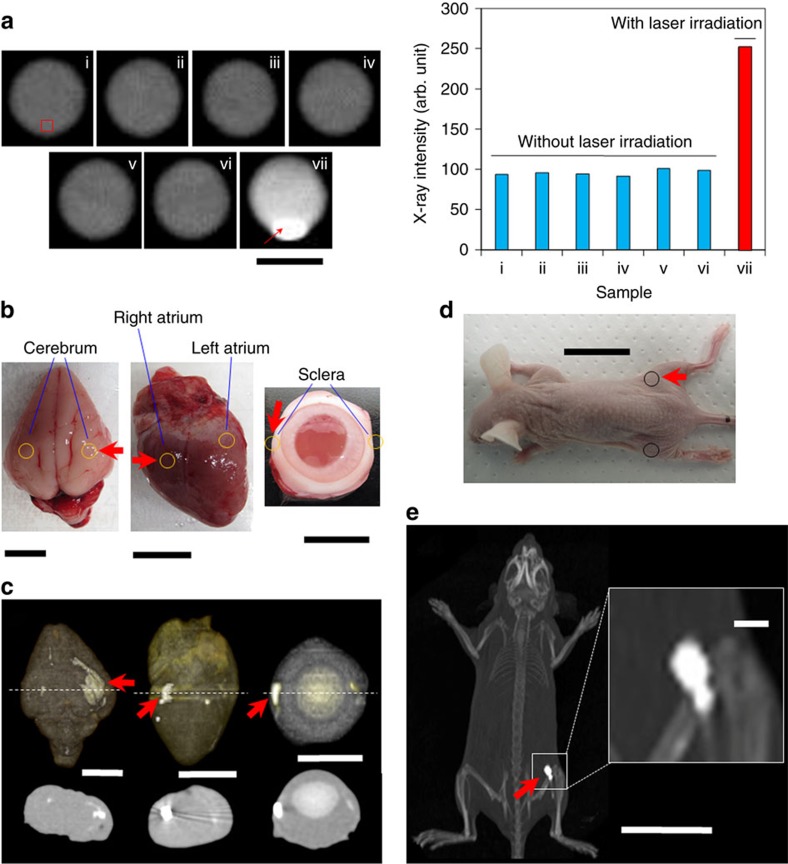
Targeting X-ray-enhanced imaging by laser-induced transformation of LM nanocapsules. (**a**) X-ray imaging and analysed intensity of LM nanocapsule solution in vials at various concentration (i: 0 mg ml^−1^; ii: 0.01 mg ml^−1^; iii: 0.1 mg ml^−1^; iv: 0.25 mg ml^−1^; v- 0.5 mg ml^−1^; vi: 1 mg ml^−1^; and vii: 1 mg ml^−1^). Only sample vii was irradiated by 785 nm laser at 1 W (∼80 mW mm^−2^) for 1 h. The red square indicates the location at which the X-ray intensity was analysed in images. The red arrow represents the precipitation that formed by laser-driven transformation of LM nanocapsules. Scale bar, 1 cm. (**b**) Photos of LM nanocapsules-injected rabbit organs (left: brain, centre: heart, and right: eye ball). Orange circles show injection parts of LM nanocapsules. Red arrows show the laser-irradiated sites. Scale bars, 1 cm. (**c**) 3D-X-ray images and sectional views of rabbit heart, brain and eye ball injected with LM nanocapsules. Red arrows display the laser-irradiated sites. Dashed lines represent location for cross-section views. Scale bars, 1 cm. (**d**) Image of LM nanocapsules-injected mouse. Black circles show injection parts of LM nanocapsules. Red arrow shows the laser-irradiated site. Scale bar, 3 cm. (**e**) 3D-X-ray image of LM nanocapsules-injected living mouse. Red arrow display the laser-irradiated site. Inlet image shows the magnified view of laser-irradiated part. Scale bar, 3 cm (Inlet: 2 mm). 3D, three-dimensional

Next, we exploited this light-triggered transformation mechanism of LM nanocapsules into biological organs for examinations of the function of LMs in more physiological atmosphere. The X-ray bioimaging is one of the most established and convenient methods to monitor the condition of a disease, but still limited in a specific environment (for example, proper molecular charge balance and optimal osmotic pressure) for utility of contrast agents^56^. In addition, X-ray contrast agents were typically applied for a passive imaging without a controlling of their contrast values via natural circulation in blood vessels or accumulation in tissues and organs. Spatiotemporal regulation of X-ray intensity and signal enhancement of contrast agents has possibility to provide more clear images to monitor complicated biological events as a next generation active imaging nanoagent^57^,^58^. To overcome the limitations and improve the properties of contrast agents, we therefore investigated the potential capability of light-active transformation behaviour of LM nanocapsules for X-ray computed tomography (CT) (Fig. 6). Interestingly, X-ray intensity of LM nanocapsules was dramatically enhanced by laser irradiation although LM nanocapsules themselves do not have strong X-ray signal in a batch due to the low density of minuscule amount LM in a tiny space of nanocapsules (Fig. 6a). In particular, the precipitation in laser-irradiated LM nanocapsule solution displayed the brightest X-ray signal. The laser-induced LM nanocapsules transformed to massive LM aggregations, as we demonstrated by optical microscopy, TEM and DLS measurements, that could be restored the original X-ray contrast property that is sufficient density for X-ray imaging. Indeed, Liu *et al*. recently reported that strong X-ray attenuation property and high density of pristine LM Ga were effective for *in vitro* X-ray imaging^18^. To further evaluate the unique optical transformation property of the LM, we then injected LM nanocapsules into various type of organs (heart, brain and eye ball) from rabbit for analysing of X-ray CT images (Fig. 6b). LM nanocapsules displayed strong X-ray signals at the targeted sites in all type of organs after laser irradiation (Fig. 6c and Supplementary Movie 8). The enhancement of X-ray contrast was also confirmed in a body of living mouse after laser irradiation (Fig. 6d,e, and Supplementary Movie 9). Therefore, we consider that transformation of LM nanocapsules could be accumulated at these targeted sites lead to enhance contrast during X-ray imaging. These results clearly show that transformation of LM nanocapsules is promising as the spatiotemporal X-ray marker in biological tissues and organs at the desired locations. To the best of our knowledge, this is the first work that shows how to change the X-ray signal from nanomaterials by light exposure. This active photodynamic imaging system would be potentially useful for development of an advanced diagnostic imaging with targeted cancer therapies in combination with the various multi-functions of LM nanocapsules.

### Elimination of cancer cells

**Figure 7:**
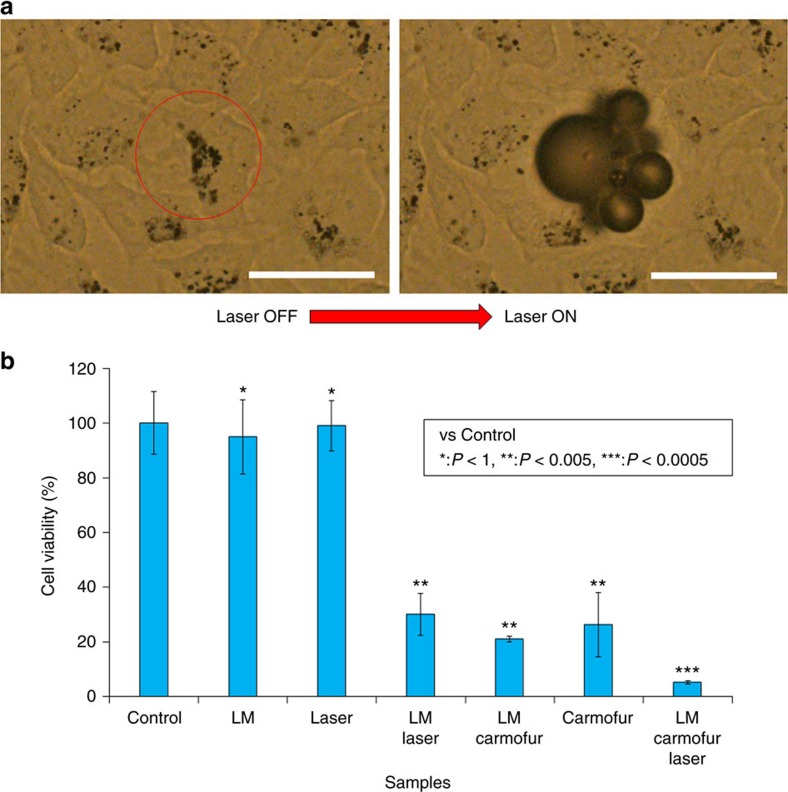
Cancer cell elimination by laser-induced LM nanocapsules. (**a**) Real-time elimination of HeLa cells caused by laser-induced LM nanocapsules before and after laser irradiation (808 nm, 564 mW, ∼287 μW μm^−2^). The red circle shows laser irradiation position and area. Scale bars, 50 μm. (**b**) Multi-dimensional cancer cell elimination using the indicated physicochemical treatments (785 nm, 1 W, ∼80 mW mm^−2^). Error bars represent s.d.'s of three separate measurements. *P<1; ^**^P<0.005; ^***^P<0.0005.

Functionalized LMs are flexible^1^,^2^, have drug-loading capability^10^ and are beneficial for human health^30^,^31^,^32^. Accordingly, their heat and ROS generation and transformation properties could be used for the destruction of cancer cells. To test this possibility, we incubated HeLa cells with 200 μg ml^−1^ LM nanocapsules for 24 h. After washing with fresh medium, cells were irradiated with an 808-nm laser (564 mW, ∼287 μW μm^−2^) for 3 s through a × 20 objective. Before laser irradiation, we confirmed that LM nanocapsules were taken up by cells, forming black aggregates (Supplementary Fig. 17). After NIR laser irradiation, bubbles formed (indicating PA properties) and floating cellular fragments were immediately observed (Fig. 7a and Supplementary Fig. 18 and Supplementary Movie 10). Control cells without LM nanocapsules did not exhibit any such visible changes. Destruction of LM-dosed cells was localized at the site of irradiation, possibly as a result of laser-induced heating and acoustic-explosion of internalized LM nanocapsules, vaporization of adjacent cellular matter and concomitant cell rupture. These results are consistent with cell viability data (Fig. 7b). Cells pre-seeded in 96-well plates were incubated with LM nanocapsules, LM nanocapsules containing carmofur or nanocapsules containing carmofur. After incubation for 24 h, cells were washed with fresh growth medium and wells were irradiated with a fibre-coupled continuous laser at 785 nm for 3 min at 1 W (∼80 mW mm^−2^). Whereas more than 95% of non-irradiated HeLa cells incubated with LM nanocapsules were viable, laser irradiation decreased cell viability to 30% because of the powerful photothermal property of LM and ROS generation from laser-induced LM. Laser irradiation itself did not effect on cell viability at all as well as LM nanocapsules themselves. Viability of nanocapsules containing carmofur was only 25%. LM nanocapsules had almost the same antitumour effect as nanocapsules containing carmofur. The most dramatic decrease in cell viability (down to 8%) was caused by LM nanocapsules containing carmofur with laser irradiation. These results clearly indicate that laser-triggered multifunctionality of LM nanocapsules, such as photothermal property, ROS generation and drug releasing, can effectively eliminate cancer cells.

### *In vivo* tumour elimination by laser-driven LM nanocapsules

**Figure 8:**
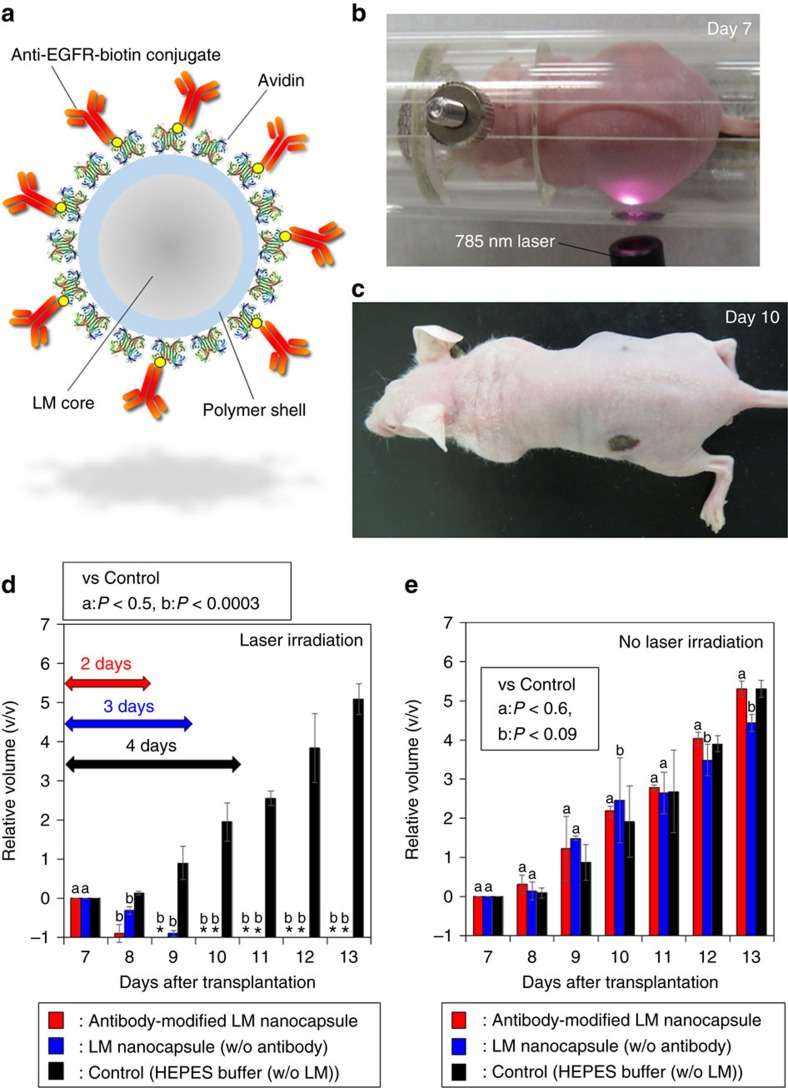
Photodynamic and hyperthermia destruction of tumours *in vivo*. (**a**) Schematic illustration of antibody-functionalized LM nanocapsules for target tumour elimination. (**b**) A mouse with large tumours on its left and right side back 7 days after tumour cell transplantation (day 7). The tumour on the left side back is being irradiated with 785 nm laser. (**c**) A mouse after 3 days of treatment (day 10) with Anti-EGFR-Biotin-Avidin-DSPE-PEG_2000_-Amine-DC(8,9)PC-LM and laser irradiation of the tumour on its left side back. (**d**) Relative volumes of tumours on the laser-induced left side back. HEPES dispersions of Anti-EGFR-Biotin-Avidin-DSPE-PEG_2000_-Amine-DC(8,9)PC-LM (red bars) or HEPES dispersions of Avidin-DSPE-PEG_2000_-Amine-DC(8,9)PC-LM (blue bars), or HEPES (black bars) were intratumourally injected and treated with 785 nm laser. Red, blue, and black arrows represent laser irradiation periods for HEPES dispersions of Anti-EGFR-Biotin-Avidin-DSPE-PEG_2000_-Amine-DC(8,9)PC-LM-, HEPES dispersions of Avidin-DSPE-PEG_2000_-Amine-DC(8,9)PC-LM-, and HEPES-injected mice, respectively. *N.D.: not determined because tumours were completely disappeared. Error bars represent s.d.'s of measurements from three mice. ^a^*P*<0.005; ^b^*P*<0.05; ^c^*P*<0.5; ^d^*P*<0.0005. (**e**) Relative volume of tumours on the right side back that were injected HEPES dispersions of Anti-EGFR-Biotin-Avidin-DSPE-PEG_2000_-Amine-DC(8,9)PC-LM (red bars) or HEPES dispersions of Avidin-DSPE-PEG_2000_-Amine-DC(8,9)PC-LM (blue bars), or HEPES (black bars) but not subjected to laser irradiation. Error bars represent s.d.'s of measurements from three mice. ^a^*P*<0.5; ^b^*P*<0.005; ^c^*P*<0.05.

The result above hinted that if LM nanocapsules can be selectively attached onto the target cancer cells with specific tumour markers, *in vivo* laser-induced LM nanocapsules can then selectively eliminate tumour without harming normal cells. To achieve the important goal, DSPE-PEG_2000_-Amine-DC(8,9)PC-LM was functionalized with the epidermal growth factor receptor (EGFR) antibody (Anti-EGFR) by avidin-biotin supramolecular interaction for recognizing and targeting tumourous cell types (Fig. 8a). EGFRs are common tumour biomarkers expressed at high levels on the surfaces of various cancer cells^59^. For the *in vivo* studies, EGFR-positive human colon adenocarcinoma HT29 cells with overexpressed EGFRs on the cell surfaces were used as a model to exploit this system and prepare solid tumour in nude mice. Laser-induced Anti-EGFR-Biotin-Avidin-DSPE-PEG_2000_-Amine-DC(8,9)PC-LM dramatically inhibited tumour growth and consequently tumours were completely disappeared after only 3 days (Fig. 8b–d). More interestingly, we could observe that volume of tumours treated with the laser-induced antibody-functionalized LM nanocapsules was visibly decreased during laser irradiation on the first day (Day 7). The dark spots were appeared on the irradiated part due to the intra-haemorrhage caused by laser irradiation and sample injection (Fig. 8c). On the other hand, laser-irradiated tumours without LM nanocapsules increased the tumour size over time. Irradiation after injection of Avidin-DSPE-PEG_2000_-Amine-DC(8,9)PC-LM decreased the tumour volume more than injection of HEPES buffer, but less than after injection of antibody-functionalized LM nanocapsules. However, the laser-induced plane LM nanocapsules without antibody required only 4 days for complete tumour disappearance. Several research groups previously reported that laser-induced nanocarbons or gold nanoparticles need period at least 1 week for cancer treatment^60^,^61^. These rapid therapeutic effects of the LM nanocapsules are probably involved in the inhibition against tumour vascularization and blood flowing by laser-induced transformation of LM nanocapsules in tumour blood vessels as observed in Fig. 5. Anyway, the LM optical technology might be contribute to quality of life for cancer regression. In addition, the selective tumour elimination suggested that anti-EGFR-functionalized LM nanocapsules were efficiently interacted with HT29 cells and could destroy cancer by powerful photothermal and ROS generation properties of LM nanocapsules. The surface temperatures of tumours treated with laser-induced LM nanocapsules with and without antibody were monitored by IR thermography (Supplementary Fig. 19). As a result, antibody-functionalized LM nanocapsule showed higher temperature increasing behaviour rather than LM nanocapsule without antibody. Laser-induced HEPES buffer in a body of mice did not significantly increase surface temperature of tumours. Without laser irradiation, the tumour volumes increased over time regardless the presence or absence of both type of LM nanocapsules with or without antibody (Fig. 8e). In spite of the strong anticancer effect of laser-driven LM nanocapsules, the body weights of the mice increased continuously during the test period, indicating that there were no side effects that caused weight loss (Supplementary Fig. 20). These results represent that photo-promoted LM nanocapsules can work as an effective *in vivo* anticancer agent with targeting function.

### Regulation of ion channel activity

**Figure 9:**
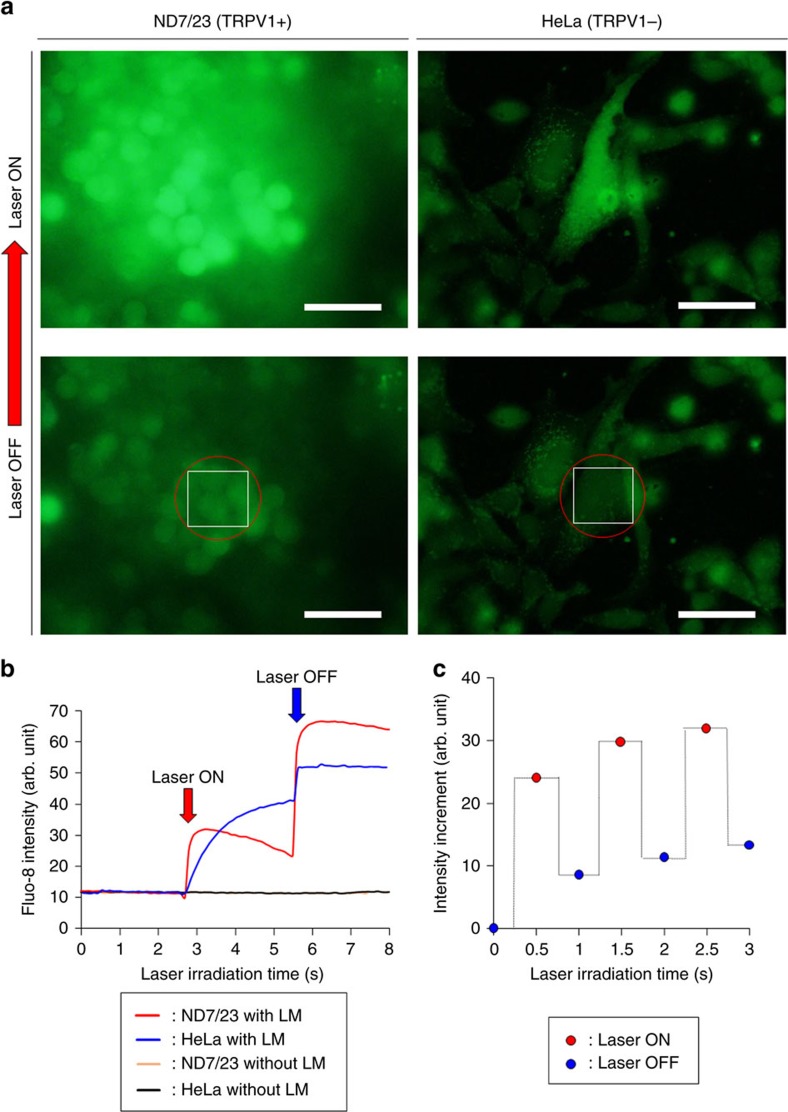
Cell stimulation by laser-induced LM nanocapsules. (**a**) Calcium influx into ND7/23 and HeLa cells when exposed to a laser source (808 nm, 133 mW, ∼68 μW μm^−2^). The irradiated site is marked with a red circle. White squares show the analysed fluorescent intensity areas. Scale bars, 50 μm. (**b**) Quantification of fluorescence intensity over time. (**c**) Response to multiple laser irradiation events.

Light stimulation is an attractive technology for investigating cell function and delivering innovative cell-based therapies^62^. However, previous techniques are invasive since a surgically implanted optical fibre or wire electrode is required to deliver poorly biopermeable light such as ultraviolet^62^, short-wavelength visible^63^ and infrared light^64^. In addition, light-active molecules suffer from slow pharmacokinetics that prevent cell activation on physiological timescales^65^,^66^. To further explore the potential biological applications of NIR laser-induced LM nanocapsules, we employed photothermal conversion efficiency and ROS generation to control ion channel activities. We decided to study the calcium ion channel because calcium transport is important for the proper functioning of organisms and cells^67^, as exemplified by neurotransmitter release, muscular contraction and enzymatic reactions. To determine whether LM nanocapsules in combination with locally generated heat and ROS were sufficient to regulate the activity of these channels, we observed intracellular calcium ion flux using the fluorescent marker Fluo-8 (Fig. 9)^68^. For this purpose, we used ND7/23 hybrid cells derived from mouse neuroblastoma and rat neuron cells. ND7/23 cells are known to overexpress the temperature-activated transient receptor potential cation channel subfamily V (TRPV1)^69^. All TRPVs are highly selective for calcium and are present on the plasma membrane or intracellular calcium storage compartments. HeLa cells were used as a control because they do not express TRPV1. Cells were pre-incubated with LM nanocapsules and Fluo-8, and then irradiated with a NIR laser. Bright green fluorescence caused by calcium influx was observed when ND7/23 cells harbouring the internalized LM nanocapsules were irradiated at 808 nm (133 mW; ∼68 mW mm^−2^; laser spot diameter: ∼50 μm) (Fig. 9a and Supplementary Movie 11). A similar but less pronounced response was observed in HeLa cells (Fig. 9a and Supplementary Movie 12). Thus, laser irradiation increased local temperature and generated large amounts of ROS, resulting in open ion channels and cytoplasmic calcium accumulation through TRPV1 or other related channels (Fig. 9b). Indeed, ROS are known to regulate ion channel activity during stress, hormone signalling, and immunological responses^70^. In addition, fast quenching and restoration of Fluo-8 fluorescence were observed at 0.5 s intervals of laser irradiation, reflecting continuous activation and silencing of the channels when the NIR laser was set to ‘ON' or ‘OFF' modes, respectively (Fig. 9c and Supplementary Movie 13). These data not only demonstrate the seamless opening and closing of channels on a microsecond timescale but also highlight the rapid stimulation of neuronal cells as a result of the fast heating and ROS generation capabilities of LM nanocapsules. We used propidium iodide staining to confirm that cell lines survived the laser treatment (Supplementary Fig. 21). In summary, these results indicate that LM nanocapsules can also act as nanostimulators for the remote control of ion channels using an external light source. We believe that one of the advantages of LM nanocapsules in comparison with other light-driven cellular stimulation^62^ including our previous research applied semiconducting polymer nanoparticles^71^ is the possibility to be excited at various wavelength of light because LMs have absorbance over a wide range in the optical region (Supplementary Fig. 22). At least, compared with ordinary photothermal materials, LM nanocapsules generally possess overwhelming superiorities of easy modification, good thermal stability, robustness against photobleaching and light-driven unique transformation property. Yet the investigation of LM nanocapsules as photothermal materials is still at an infancy stage. In these regards, developing LM photothermal materials is highly desired for various applications. Furthermore, the LM nanocapsules have stronger stimulation activity for various cell type by the synergy effect from both of photothermal property and ROS generation rather than polymer nanoparticles^71^, nanocarbons^26^ and Au-NRs^72^ that can mainly produce not ROS but heat. Furthermore, capacities of drug-loading and high biocompatibility of the LM nanocapsules might be useful to develop an innovative nanostimulator.

### PA imaging using NIR-stimulated LM nanocapsules

**Figure 10:**
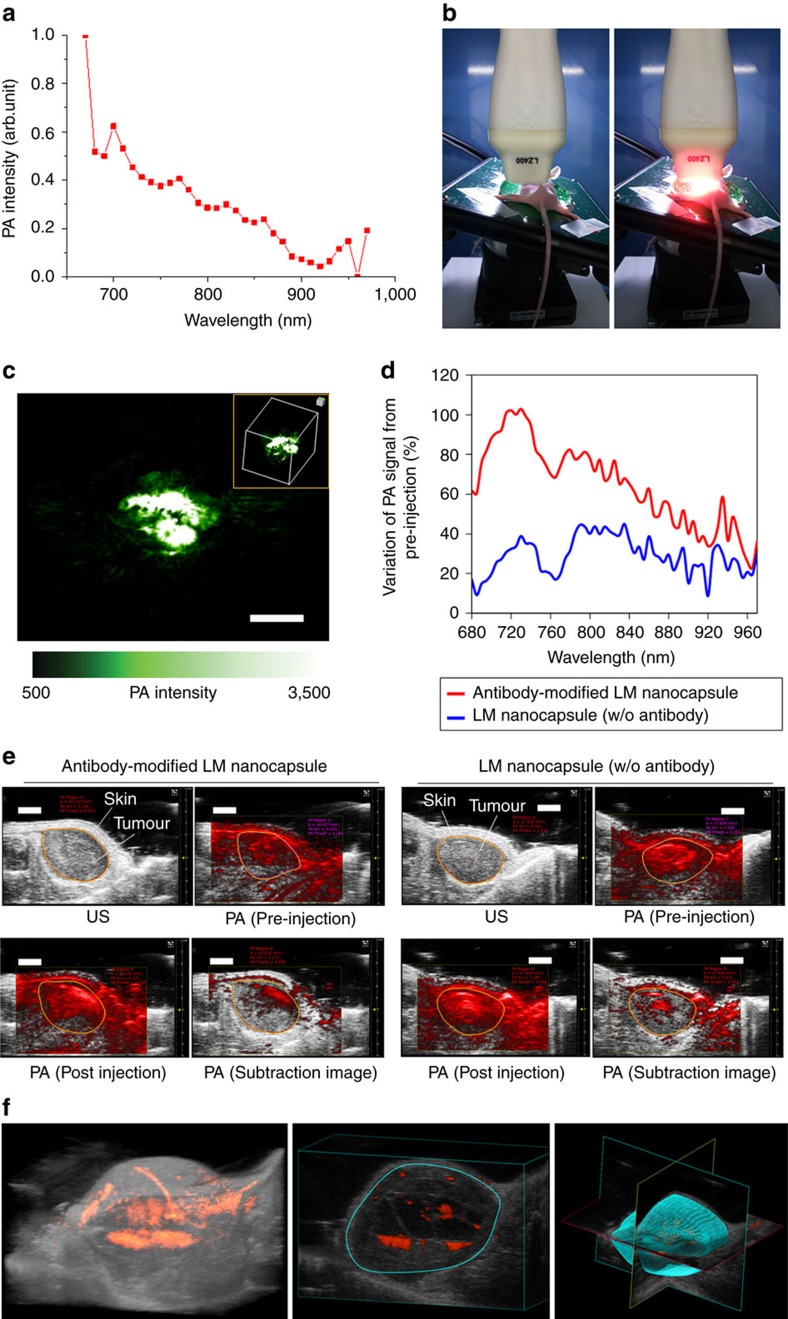
*In vivo* PA imaging using LM nanocapsules. (**a**) PA intensity of LM nanocapsules (100 μg ml^−1^) at different wavelength. (**b**) Photos of *in vivo* PA imaging set-up before (left) and after laser exposure (right). (**c**) PA (green) image of LM nanocapsules (100 μg ml^−1^) under a mouse's skin. Excitation wavelength of laser is 680 nm. Inlet shows the 3D image of subcutaneous localization of LM nanocapsules. Scale bar, 4 mm. (**d**) Enhancement of PA intensity in tumour by antibody-functionalized LM nanocapsules (100 μg ml^−1^) in wide wavelength range for laser excitation. Variations of PA signals were calculated as post-injections minus pre-injections. (**e**) Antibody-functionalized LM nanocapsules (100 μg ml^−1^) targeted tumour imaging in living mice. Ultrasound (US) (grey) and PA (red) images were taken through the tumour by 750 nm laser excitation. Orange circles show the analysing parts of the PA signal for Fig. 8d. Scale bars, 2 mm. (**f**) 3D imaging of tumour treated by antibody-functionalized LM nanocapsules. Excitation wavelength of laser is 750 nm. Blue circle shows the part for construction of 3D structure. 3D, three-dimensional

Optically active multifunctional nanomaterials promise to advance a range of biophotonic techniques through nanoscale optical effects and integration of multiple imaging and therapeutic modalities^73^,^74^,^75^,^76^. The heating and acoustic-explosion properties of laser-induced LM nanocapsules also suggest both therapeutic (cell destruction and activation) and detection (PAs) potential. To assess the latter *in vivo*, we first measured the PA signal of LM nanocapsules at wavelengths of 670–970 nm (Fig. 10a). We found that the highest signal was obtained at 680 nm and that PA intensity increased with the concentration of LM nanocapsules (Supplementary Fig. 23). In addition, LM nanocapsules have stronger PA intensity over a wide excited wavelength range rather than Au-NR2 probably due to the high photothermal conversion efficiency and high aqueous stability of LM nanocapsules (Supplementary Fig. 24).

We then injected subcutaneously the lower back of a mouse with LM nanocapsules (100 μg ml^−1^) or distilled water. PA imaging set-up is illustrated in Fig. 10b. We found that water alone did not generate a strong PA signal; however, LM nanocapsules produced neatly contrasted PA images (Fig. 10c). We also observed a linear correlation between LM concentration and the corresponding PA signal (Supplementary Fig. 25). More interestingly, mice injected with Anti-EGFR-functionalized LM nanocapsules showed a significant enhancement of PA signal (∼22–102%) in the tumour from pre-injection at wide wavelength range (680–970 nm) compared to the control tumour model injected with plain LM nanocapsules (∼9–45%) (Fig. 10d,e, and Supplementary Fig. 26). Three-dimensional-imaging also showed that antibody-functionalized LM nanocapsules distributed in tumours (Fig. 10f). Therefore, we concluded that LM nanocapsules could be a useful LM concentration-dependent PA contrast agent in living mice and antibody-functionalized LM might be effective as theranostic nanoparticle for the targeting tumour detections and eliminations.

## Discussion

In this study, we described the design and application of NIR laser-stimulated LM nanocapsules. Upon ultraviolet irradiation, the core of LM nanocapsules was further stabilized by intramicellar crosslinking between neighbouring DSPE-PEG_2000_-Amine and DC(8,9)PC moieties. The encapsulated LMs in polymer micelles are water-soluble with high biocompatibility, and amenable to transformation by NIR laser irradiation. Moreover, we show that NIR laser-stimulated nanocapsules can be used to effectively eliminate cancer cells due to their photothermal properties, ROS generation, controlled release of anticancer drugs, optical manipulations of a microfluidic blood vessel model, and targeted marking for X-ray-enhanced imaging in biological organs. Intercellular Ca^2+^ flux could also be spatiotemporally controlled using LM nanocapsules. Furthermore, nanocapsules remotely produced PA signals in mice when irradiated with a NIR laser. This study is the first to exploit the powerful physicochemical properties of LM nanomaterials for cancer cell elimination, spatiotemporal remote control of cell activity *in vitro*, and PA imaging in living mice.

These attractive multifunctionality of LM nanocapsules are promising for further *in vivo* theranostic applications as well as other general NIR sensitive nanomaterials. In particular, laser-induced unique transformations of LM nanocapsules would offer important opportunities for future practical applications, such as low-power CW laser welding technology, reconnecting of the transected electronics, non-contact wiring on electrodes of flexible diagnostic devices, soft machine manufacture, locomotion-assisted devices, microfluidic valves and pumps or artificial nanorobots^1^,^2^,^3^,^4^,^5^,^6^,^7^,^8^,^9^,^10^ in addition to optical manipulations of a blood vessel and *in vivo*-targeted X-ray marking as we described in this work. Furthermore, high biocompatibility of LM nanocapsules will be essential for construction of future biomedical strategies to improve biosafety and biosecurity of nanomaterials. Meanwhile, chemotherapy is one of the principal modes of treatment for cancer, but the effectiveness of chemotherapy is limited by anticancer drug resistance^77^. Our light-driven LM technology has possibility to provide multidimensional cancer theranostics that might be more effective against the cancer drug resistance. The multi-functionality of light-driven LM is also useful for chemical and biomedical engineering applications. Accordingly, chemically functionalized LM nanocomplexes have great potential for the development of new therapeutic and diagnostic tools for cancer treatment, cell stimulation, and bioimaging applications.

The findings described here also provide a valuable technology with potential applications in various fields of biology, including cancer research, brain science and immunology. This knowledge could be, especially, very useful for the development of nanoscale stimulators, such as power sources for deep brain stimulation and optical biotelemetry, and the creation of unique cell therapies and tissue engineering. Finally, we believe the dramatic morphological changes to LM triggered by NIR irradiation could also be used for light-induced wiring on electrical devices^48^,^49^ and light-driven self-healing materials^50^ in addition to the improvement of biomedical theranostic effects for various disease treatment and care.

## Methods

### Synthesis of LM nanocapsules

LM (Ga:In=75.5:24.5 wt%; Alfa Aesar, Ward Hill, MA, USA) (10 mg), DSPE-PEG_2000_-Amine (DSPE-020PA; Sunbright, NOF) (10 mg) and DC(8,9)PC (Avanti Polar Lipids, Alabaster, AL, USA) (2 mg) were mixed in distilled water (10 ml) by pulse-type sonication (VCX-600; Sonics, Danbury, CT, USA) for 10 min. The mixture was then irradiated at 254 nm for 2 h with three hand-held ultraviolet lamps (SLUV-4; AS ONE, Osaka, Japan), followed by centrifugation at 1,000 r.p.m. for 10 min at 20 °C (3,740; Kubota, Tokyo, Japan). The supernatant thus obtained (LM concentration: ∼100 μg ml^−1^) was used for further structural and optical characterization. Meanwhile, a concentrated solution of LM nanocapsules (1, 10, 100 and 320 mg ml^−1^) was synthesized without centrifugation and was then used for *in vitro* experiments.

LM nanocapsules containing carmofur were prepared as follows. Briefly, carmofur (5 mg) was added to a mixture of LM (10 mg), DSPE-PEG_2000_-Amine (10 mg) and DC(8,9)PC (2 mg). After pulse sonication for 10 min, the solution was ultraviolet-irradiated for 2 h at 254 nm and finally centrifuged at 1,000 r.p.m. for 5 min to remove water-insoluble carmofur. Control carmofur without LM was synthesized with DSPE-PEG_2000_-Amine and DC(8,9)PC only according to the above protocol.

DSPE-PEG_2000_-Amine-SWCNT and DSPE-PEG_2000_-Amine-MWCNT were synthesized as follows: SWCNT (CoMoCAT CG300, purity>95%, average diameter and length: 0.6–1.1 nm and 0.1–1.0 μm, respectively; SouthWest NanoTechnologies, Norman, OK, USA) (10 mg) or MWCNT (Nanocyl 3,100, purity>95%, average diameter 9.5 nm, average length 1.5 μm; Nanocyl, Belgium) (10 mg) were mixed with DSPE-PEG_2000_-Amine (10 mg) in water (10 ml) for 10 min by pulse sonication. These solutions were used in cell viability assays.

Anti-EGFR-Biotin-Avidin-DSPE-PEG_2000_-Amine-DC(8,9)PC-LM was synthesized as follows. DSPE-PEG_2000_-Amine-DC(8,9)PC-LM (10 mg ml^−1^) was mixed with avidin from egg white (1 mg) (Wako, Osaka, Japan) and *N*-hydroxysuccinimide (Wako) (10 mg) and water-soluble carbodiimide (Wako) (10 mg) in distilled water for overnight. Unreacted molecules were removed by centrifugation at 15,000 r.p.m. for 10 min at 20 °C and washed three times by water. After redispersing into HEPES buffer (0.1 M, pH=7.3), the avidin-functionalized DSPE-PEG_2000_-Amine-DC(8,9)PC-LM (Avidin-DSPE-PEG_2000_-Amine-DC(8,9)PC-LM) (10 mg ml^−1^, 10 ml) was reacted with biotin-conjugated EGFR monoclonal antibody (Anti-EGFR-Biotin) (50 μg ml^−1^, 250 μl) (Thermo Fisher Scientific, Waltham, MA, USA) via avidin-biotin supramolecular interaction. Concentration of avidin and EGFR antibody on DSPE-PEG_2000_-Amine-DC(8,9)PC-LM (10 mg ml^−1^) were estimated to 321 μg ml^−1^ and 1.2 μg ml^−1^, respectively, by Pierce 660 nm protein assay kit (Thermo Fisher Scientific).

### Characterization of LM nanocapsules

The structure and morphology of the prepared LM nanocapsules were observed using high-resolution TEM (EM-002B; Topcon, Tokyo, Japan) with an accelerating voltage of 120 kV. A small droplet of sample placed on a grid or 10 μl of sample solution (LM concentration: 100 μg ml^−1^) were irradiated using a fibre-coupled CW laser at 785 nm for 3 min (spot diameter, ∼4 mm; maximum power: 1 W, ∼80 mW mm^−2^; BRM-785-1.0-100-0.22-SMA; B&W Tek, Newark, DE, USA). The polymer shell structure of nanocapsules and STEM/EDS mapping were performed by Nanoscience Co. in Evans Analytical Group Company, Inc. (Tokyo, Japan). The samples were imaged with a FEI Tecnai TF-20 FEG/TEM operated at 200 kV in bright-field TEM mode, high-resolution (HR) TEM mode, and high-angle annular dark-field (HAADF) STEM mode. The STEM probe size was 1–2 nm nominal diameter. EDS mapping were acquired on Oxford INCA, Bruker Quantax EDS system.

The hydrodynamic diameter of LM nanocapsules was examined by DLS (Photal FPAR-1,000; Otsuka Electronics, Osaka, Japan). DLS diagram of laser-induced LM nanocapsules was also measured. A 100 μl of sample solution (LM concentration: 100 μg ml^−1^) was irradiated using a fibre-coupled CW laser (maximum power: 1 W, ∼80 mW mm^−2^) at 785 nm for 1 h before DLS measurements.

The concentration of LM and carmofur in nanocapsules was estimated with a ultraviolet–visible–NIR spectrophotometer (V-730 BIO; Jasco, Tokyo, Japan).

### Cell viability test

Human cervical cancer (HeLa) cells (EC84121902-F0; DS Pharma Biomedical, Tokyo, Japan) were pre-seeded in 96-well plates at 3 × 10^4^ cells well^−1^ in 200 μl of RPMI 1,664 (Gibco, Carlsbad, CA, USA) and incubated for 24 h. Cells were then incubated with DSPE-PEG_2000_-Amine-DC(8,9)PC-LM, DSPE-PEG_2000_-Amine-SWCNT, DSPE-PEG_2000_-Amine-MWCNT, methyl-terminated-hydrophilic polymer-conjugated Au-NR (average diameter: 10 nm; average length: 41 nm; peak Surface plasmon resonance wavelength: 808 nm;<0.0001% CTAB; Sigma-Aldrich, St Louis, MO, USA), and methoxy-PEG_2000_-SH modified Au-NR (average diameter: 9 nm; average length: 29 nm; peak Surface plasmon resonance wavelength: 770 nm; Cytodiagnostics) at nine different concentrations (0, 25, 50, 100, 200, 400, 800, 1,200 or 1,600 μg ml^−1^) for 24 h. After washing with fresh medium, cells were incubated with the cell proliferation reagent WST-1 (Roche Diagnostics, Manheim, Germany) according to the manufacturer's instructions. Sample absorbance was measured at 450 nm using a microplate reader (Model 680; BIO-RAD, Hercules, CA, USA); the reference wavelength was 620 nm. Statistical analyses were performed using an analysis of variance with Tukey's test (analysis of variance (ANOVA)), and a *P* value of less than 0.05 was considered significant.

### *In vivo* viability test

All animal experiments were performed strictly in accordance with protocols approved by the Institutional Animal Care and Use Committee of AIST, NTU, Shin Nippon Biomedical Laboratories, and Primetech. Five-week-old female mice (Average weight=15 g, *N*=5; C57BL/6NCrSlc; Japan SLC, Shizuoka, Japan) were anaesthetized with isoflurane (2 l min^−1^) via an automated inhalation anaesthesia apparatus (KN-1,071; Natsume, Tokyo, Japan). Suspensions (300 μl; LM nanocapsule and PBS buffer; LM concentration=10 and 320 mg ml^−1^) were injected into caudal vein of mice. Following the injections of LM nanocapsules (LM nanocapsule in distilled water and PBS buffer without LM nanocapsules), the viability and body weight of mice were carefully checked for 19 days.

### Cellular uptake of LM nanocapsules

Internalization and distribution of LM nanocapsules in cancer cells were analysed using confocal laser microscopy (LSM 5 PASCAL; Carl Zeiss, Oberkochen, Germany). HeLa cells were pre-seeded in 35 mm glass-bottom dishes (Iwaki, Tokyo, Japan) and incubated for 24 h before the experiment, washed with phosphate-buffered saline (PBS; Gibco), and incubated with fresh RPMI containing 100 μg ml^−1^ LM nanocapsules for 24 h. Cells were then washed twice with PBS and mounted in fresh PBS for observation.

### Laser-induced temperature increase

A droplet of LM (1 mg) was gently placed on a filter paper (Advantec, Tokyo, Japan) with a volumetric pipette and directly irradiated using a fibre-coupled CW laser at 785 nm for 5 min at maximum power (1 W, ∼80 mW mm^−2^). The surface temperature of a LM droplet was monitored by IR thermography (i7; FLIR, Nashua, NH, USA). Temperature increase of laser-induced LM suspensions was investigated as follows. LM nanocapsules in distilled water (300 μl, LM concentration: 100 μg ml^−1^) were added to a dispersible plastic cuvette (Ocean Optics, Dunedin, FL, USA). Samples were irradiated using a NIR laser for 5 min. Irradiated distilled water without nanocapsules was used as a control. The real-time temperature of the solutions (not directly under the laser beam) was measured using a temperature sensor (AD-5601A; A&D, Tokyo, Japan). Statistical analyses were performed using an analysis of variance with Tukey's test (ANOVA), and a *P* value of less than 0.05 was considered significant. Au-NR1 or Au-NR2 (300 μl, Au-NR concentration: 100 μg ml^−1^) was also irradiated in a similar procedure as the LM nanocapsules to examine the laser-induced temperature changing and photothermal conversion efficiency.

Photothermal conversion efficiency of LM nanocapsules was determined according to the previous methods^78^,^79^,^80^. Detailed calculation was given as following:




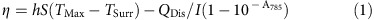




where *h* is the heat transfer coefficient, *S* is the surface area of the container and the value of *hS* is obtained from the equation (4) and Fig. 3b. The maximum steady temperature (*T*_max_) of the solution of the LM nanocapsules was 51.8 °C and environmental temperature (*T*_Surr_) was 21.5 °C. So, the temperature change (*T*_Max_−*T*_Surr_) of the solution of the LM nanocapsules was 30.3 °C. The laser power *I* is 1 W. The absorbance of the LM nanocapsules at 785 nm *A*_785_ is 0.122. *Q*_Dis_ expresses heat dissipated from the light absorbed by the solvent and container.

In order to gain *hS*, a dimensionless parameter *θ* is introduced as followed:




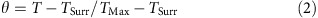




A sample system time constant *τ*_s_ can be calculated as equation (3).




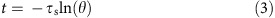




According to Fig. 3b, *τ*_s_ was determined and calculated to be 122.2 s.




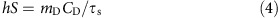




In addition, *m*_D_ is 0.3 g and *C*_D_ is 4.2 J g^−1^ °C^−1^. Thus, according to equation (4), *hS* is deduced to be 10.31 mW °C^−1^.

*Q*_Dis_ expresses heat dissipated from the light absorbed by the plastic cell itself, and it was measured independently to be 185.6 mW using a dispersible plastic cuvette cell containing distilled water.

Thus, substituting according values of each parameter to equation (1), the 785-nm laser heat conversion efficiency (*η*) of the LM nanocapsules can be calculated to be about 52%. Photothermal conversion efficiency of commercial Au-NR1 was also analysed as a control from the laser-induced temperature changings (Supplementary Fig. 8). The *τ*_s_ of the Au-NR1 was determined and calculated to be 117.8 s, *hS* is deduced to be 10.69 mW °C^−1^. The absorbance of the Au-NR1 at 785 nm *A*_785_ is 2.443, then the 785-nm laser heat conversion efficiency (*η*) of the Au-NR1 can be calculated to be about 17% that value is almost closed to previous reports^78^,^79^,^80^.

Photothermal stability of LM was performed as following way. A droplet of LM (10 mg) or ICG powder (Wako) (10 mg) was irradiated at 785 nm for 1 h at maximum power (1 W, ∼80 mW mm^−2^) in a vial. Laser-induced LM (10 mg) was dispersed in distilled water (10 ml) with DSPE-PEG_2000_-Amine (10 mg) by pulse sonication for 10 min. Meanwhile, laser-irradiated ICG (1 mg) was directly dissolved in distilled water (10 ml) without functionalization by DSPE-PEG_2000_-Amine. After 20 and 60 times dilution with water for each samples, respectively, optical absorbance spectra were analysed by ultraviolet–visible–NIR spectrometer.

Effect of laser wavelength on temperature increasing behaviour of various samples (LM nanocapsules, Au-NR1, and ICG) was investigated using a 670-nm laser (spot diameter, ∼5 mm, maximum power: 300 mW, ∼15 mW mm^−2^; BWF-670-300E; B&W Tek, Newark, DE, USA) and a 1,064 nm laser (spot diameter∼2 mm; maximum power: 1 W, ∼320 mW mm^−2^; BL106-C; Spectra Physics, Santa Clara, CA, USA) in addition to a fibre-coupled continuous 785 nm laser (spot diameter, ∼4 mm; maximum power: 1 W, ∼80 mW mm^−2^). Each material was dissolved in milliQ water (Concentration: 100 μg ml^−1^). Sample (300 μl) in a plastic cuvette was irradiated using a NIR laser for 5 min.

### Thermal and freezing resistances of LM nanocapsules

Thermal and freezing stabilities of LM nanocapsules was determined as follows. The LM nanocapsules solution (500 μg ml^−1^) was heated at 80 °C for 3 h in an oven (MOV-450S; AS ONE, Osaka, Japan) or frozen in a deepfreezer (MDF-C8V1-PJ; Panasonic, Osaka, Japan) at −80 °C for 1 h. Au-NR1 (500 μg ml^−1^) was also heated and frozen the same way as the LM nanocapsules. Optical absorbance spectra were analysed by ultraviolet–visible–NIR spectrometer after 10 times dilution with water for each sample.

### Thermal expansion of LMs

Thermal expansion of LMs was investigated as follows. The thermometer of Galinstan (Geratherm Medical AG, Geschwenda, Germany) was directly irradiated using a fibre-coupled CW laser at 785 nm for 3 min at maximum power (1 W, ∼80 mW mm^−2^).

The LM droplet (20 μl) was put on a slide glass and then heated (not directly to the LM) by a gas burner for 3 min. The surface temperature of a LM droplet was also monitored by IR thermography.

LM nanocapsules (LM=100 μg ml^−1^, DSPE-PEG_2000_-Amine=1 mg ml^−1^, DC(8,9)PC=1 mg ml^−1^) and ultrviolet-crosslinked polymer nanocapsules without LMs (DSPE-PEG_2000_-Amine=1 mg ml^−1^, DC(8,9)PC=1 mg ml^−1^) water suspensions were heated at 130 °C for 30 min in glass test tube. The size of these nanocapsules were determined by DLS before and after heating. The structure of heated LM nanocapsules was also observed by TEM.

### ROS detection

ROS generated in enzyme-coupled reactions were detected by H2DCFDA (Molecular probes, Thermo Fisher Scientific) in a 96-well plate with black walls and a clear bottom (Thermo Fisher Scientific). LM nanocapsules (100 μl well^−1^, LM concentration: 100 μg ml^−1^) were incubated with HRP (50 μl well^−1^, stock solution 20 μg ml^−1^; Wako) and H2DCFDA (50 μl well^−1^, stock solution 20 μM) for less than 10 min at 20 °C. Samples were then irradiated with a NIR laser for 3 min at 1 W. PBS without nanocapsules was used as a control. Green fluorescence derived from ROS generation was detected with a fluorescence microplate reader (InfiniteF200 PRO; Tecan, Männedorf, Switzerland) at 485 nm excitation and 535 nm emission wavelengths. Statistical analyses were performed using an analysis of variance with Tukey's test (ANOVA), and a *P* value of less than 0.05 was considered significant.

### ROS-involved degradation of LM nanocapsules

The processes used for H_2_O_2_- and HRP oxidation-based degradations of LM nanocapsules were similar ways to those described previously for nanocarbons^45^,^46^,^47^. The LM nanocapsules were dispersed in water at a concentration of ∼1 mg ml^−1^; this dispersion (0.3 ml) was mixed with PBS (0.7 ml) containing H_2_O_2_ (10 μl of 80 mM). For enzymatic degradation of LM nanocapsules, HRP (50 μg; Wako) was added the reaction media. The final concentration of H_2_O_2_ in the LM suspension was approximately 800 μM. The reaction mixture was maintained at 37 °C for 3 days. To replenish their levels, additional HRP (50 μg) was added after 4 h and additional H_2_O_2_ (10 μl of 80 mM) was added after 4, 8, 24 and 48 h. For investigation of H_2_O_2_-treatment on LM nanocapsules, HRP was not added to the reaction. The amount of LMs in the reaction mixture was estimated based on light absorbance at 700 nm using ultraviolet–visible–NIR spectrometer.

### Controlled release of carmofur

The DSPE-PEG_2000_-Amine-DC(8,9)PC-LM encapsulating carmofur solution (LM: 200 μg ml^−1^, carmofur: 250 μg ml^−1^) was placed in a 96-well plate (200 μl well^−1^) and irradiated with a fibre-coupled CW laser at 785 nm for 30 min at maximum power (1 W, ∼80 mW mm^−2^). The collected solution (total volume, 0.5 ml) from three samples subjected to laser induction was centrifuged using a centrifugal filter unit (Ultrafree-CL microcentrifuge filter with Durapore polyvinylidene fluoride membrane, pore size: 0.1 μm; Millipore, Billerica, MA, USA) at 12,000*g* for 4 min at 20 °C. Absorbance of the released carmofur was measured three times using a ultraviolet–visible–NIR spectrophotometer. A sample not subjected to laser irradiation (0.5 ml) was also centrifuged and the concentration of spontaneously leaked carmofur from LM nanocapsules was detected with a spectrophotometer. Carmofur released from LM nanocapsules by laser irradiation was estimated as the difference in concentration (38 μg ml^−1^) before and after laser treatment.

### Microfluidic devices

The microdevice based on straight microchannels (width: 100 mm, depth: 50 mm) and fabricated by soft lithography and photolithography methods. First, the PDMS (Sylgard 184; Dow Corning, Midland, MI, USA) base and crosslinker (Sylgard 184; Dow Corning) were mixed at a weight ratio of 10:1, as specified by the manufacturer. Second, the mixture was subjected to shear mixing to ensure the uniform blending of the host matrix and the crosslinker. Next, the mixture was degassed in vacuum. A master was prepared by exposing and developing a photoresist pattern on a silicon wafer (SU-8 50; MicroChem, Westborough, MA, USA). The PDMS/crosslinker mixture was poured onto the master. After curing, the PDMS substrate was peeled away from the master. Then, the PDMS sheet was punched with holes (diameter: 3 mm) to produce reservoirs. The PDMS and glass (Matsunami Glass, Osaka, Japan) substrates were closely bonded to each other by plasma treatment using a vacuum plasma coater (PDC210; Yamato Scientific, Tokyo, Japan). Finally, the microchannels were hydrophilized using a water-soluble polymer (lipidure CR1705; NOF). The lipidure was diluted 50 times with ethanol and introduced into the microchannels with a syringe or by decompression treatment. After several minutes, the microchannels were dried using a syringe.

### Observation of laser-induced LM nanocapsules transformation in a microchannel

The transformation of LM nanocapsules and the temperature distribution in the microchannel using the laser irradiation set-up were performed as follows. The general procedures for these experiments are illustrated in Fig. 5a. An 808-nm 254 mW (∼129 μW μm^−2^) NIR laser beam from a CW diode laser (Sigma Koki, Tokyo, Japan) was incorporated in a microscopy system (IX73; Olympus, Tokyo, Japan). The laser beam (laser spot diameter: 50 μm) was focused on the target position with the objective (× 20 magnification; aperture 0.75; UPLSAPO20X, Olympus) at 20 °C. Images were recorded using an electron multiplying-charge coupled device (EM-CCD) camera system (DP80, Olympus) before and during irradiation.

The temperature distribution around the laser spot was determined from the fluorescence intensity distribution of rhodamine B (Wako) molecules on the liposomes. The control experiment for the temperature assay was also studied by introducing the distilled water including rhodamine B (1 mg ml^−1^) without the LM nanocapsules (100 μg ml^−1^) in the microchannel with a syringe. The fluorescence intensities of rhodamine B were analysed by ImageJ (Fiji). The sequential temperature controlling experiment was performed by manual laser ‘ON–OFF' switching.

### X-ray imaging

The transformation behaviour of injected LM nanocapsules in vial, organs, and living mice was analysed by Shin Nippon Biomedical Laboratories, Ltd. (Kagoshima, Japan) using the X-ray CT instrument (Asteion/S4; Toshiba Medical Systems, Tochigi, Japan). LM nanocapsule suspension (50 μl; LM concentration=50 mg ml^−1^) was injected into commercially available organs (heart (J405; *N* =2), brain (J401; *N*=2), and eye ball (J404; *N*=2) from rabbit) (Funakoshi, Tokyo, Japan). Each organ was irradiated by 785 nm laser at maximum power (1 W, ∼80 mW mm^−2^) for 5 min. Before X-ray CT observations, hearts and brains were fixed with 10% formalin natural buffer solution. On the other hand, eye balls were fixed by mixed solution including 3% glutaraldehyde and 2.5% formalin. The X-ray intensity was characterized by ImageJ. For *in vivo* imaging, LM nanocapsule aqueous suspensions (20 μl; LM concentration=10 mg ml^−1^) were injected under skin of both of thighs of 5-week-old female nude mice (Average weight=15 g, *N*=3; BALB/cSlc-nu/nu, Japan SLC). Only one side of the injected part was then irradiated by 785-nm laser at maximum power (1 W, ∼80 mW mm^−2^) for 5 min under anaesthesia before X-ray CT measurements.

### Laser-induced cytotoxicity of LM nanocapsules

Real-time monitoring of cancer cell elimination using an optical microscope equipped with a laser irradiation set-up was performed as follows. HeLa cells were pre-seeded in 35-mm glass-bottom dishes (Iwaki) for 24 h, then washed with PBS, and incubated in fresh medium containing 200 μg ml^−1^ LM nanocapsules for 24 h. Subsequently, cells were washed twice with PBS and mounted in fresh PBS for observation. The same laser irradiation set-up as transformation of LM in microfluidic devices was used for this experiments.

To assess cell viability with the WST-1 assay, HeLa cells were pre-seeded in 96-well plates at 3 × 10^4^ cells well^−1^ for 24 h. Cells were treated with samples (DSPE-PEG_2000_-Amine-DC(8,9)PC-LM encapsulating carmofur, DSPE-PEG_2000_-Amine-DC(8,9)PC-LM without carmofur or DSPE-PEG_2000_-Amine-DC(8,9)PC encapsulating carmofur), irradiated with a fibre-coupled CW laser at 785 nm for 3 min at maximum power (1 W, ∼80 mW mm^−2^), and incubated for 24 h. The following day, cells were washed with fresh RPMI, and viability was analysed using the WST-1 assay. Concentrations of LM and carmofur were adjusted to 200 and 250 μg ml^−1^, respectively. Statistical analyses were performed using an analysis of variance with Tukey's test (ANOVA), and a *P* value of less than 0.05 was considered significant.

### *In vivo* phototherapy

HT29 cells from human colon adenocarcinoma (EC91072201-G0; DS Pharma Biomedical, Tokyo, Japan) were cultured in monolayer at 37 °C in a humidified atmosphere with 5% CO_2_. The cell culture medium was McCoy's 5a (DS Pharma Biomedical) supplemented with 10% fetal bovine serum (American Type Culture Collection, Manassas, VA, USA), 2 mM Glutamine and 0.1% penicillin-streptomycin (Gibco). Five-week-old female nude mice (Average weight=15 g, *N*=9; BALB/cSlc-nu/nu, Japan SLC) were bilaterally implanted with Matrigel (Corning, NY, USA)-cell culture media (1:1 volume ratio) including HT29 cells (1 × 10^6^) via subcutaneous injection into each side back. Seven days after implantation, the mice were separated into three groups (three mice per group), and the tumours were injected with 200 μl of HEPES or 200 μl of the HEPES dispersions of Anti-EGFR-Biotin-Avidin-DSPE-PEG_2000_-Amine-DC(8,9)PC-LM, or 200 μl of the HEPES dispersions of Avidin-DSPE-PEG_2000_-Amine-DC(8,9)PC-LM. The concentration of LM was set to be 10 mg ml^−1^. From day 7, the tumours on the left side backs only were irradiated every 24 h with the 785-nm laser at maximum power (1 W, ∼80 mW mm^−2^). Because the tumours were much larger than the spot size of the laser (∼4 mm), we irradiated each tumour in five locations (3 min per location) for a total of 15 min of irradiation. The only exception was the first day, when we irradiated only one spot per tumour for 15 min. We recorded tumour size for each mouse during the treatment; i.e., for the 10 days of treatment and for two additional days once treatment was over. The size of the tumour was measured at the widest point (*W*) and along the corresponding perpendicular length (*L*). The formula for computing tumour volume (*V*) was for a standard volume of an ellipsoid, where *V*=4*π*/3 (length/2 × width/2 × depth/2). Assuming that depth equals width and *π* equals 3, *V*=1/2 × *L* × *W*^2^. The relative tumour volume was calculated as (tumour volume on the day of measurement)/(volume of the tumour on the day 7). On day 7, the average tumour volume was about 174 mm^3^. When the tumours disappeared, the tumour volumes were recorded as ‘zero'. Statistical analyses were performed using an analysis of variance with Tukey's test (ANOVA), and a *P* value of less than 0.05 was considered significant.

The surface temperatures of tumours treated with laser-induced LM nanocapsules with and without antibody were monitored by IR thermography. Temperature of laser-induced HEPES buffer-injected tumours were also analysed by thermographic camera as a control. Sample-injected mice were incubated for 15 min before the experiments. The tumours were injected with 200 μl of HEPES or 200 μl of the HEPES dispersions of Anti-EGFR-Biotin-Avidin-DSPE-PEG_2000_-Amine-DC(8,9)PC-LM, or 200 μl of the HEPES dispersions of Avidin-DSPE-PEG_2000_-Amine-DC(8,9)PC-LM. The concentration of LM was set to be 10 mg ml^−1^.

### Laser-induced cell stimulation

HeLa and ND7/23 hybrid cells derived from mouse neuroblastoma and rat neuron cells (EC92090903-F0; DS Pharma Biomedical) cells (3 × 10^4^ cells per ml) were pre-seeded in 35-mm Poly-L-lysine-coated four-well glass bottom dishes (Matsunami Glass) and incubated for 24 h. ND7/23 cells were cultured in Dulbecco's modified Eagle's medium with 200 mM glutamine, whereas HeLa were incubated in RPMI. Both media contained also 10% fetal bovine serum and 1% penicillin/streptomycin. The day before the experiments, cells were washed and incubated with DSPE-PEG_2000_-Amine-DC(8,9)PC-LM complexes (LM concentration: 200 μg ml^−1^). Control cells did not contain any nanoproducts. After 24 h, cells were washed, fresh growth medium (100 μl per well^−1^) and Fluo-8 working solution (100 μl per well) (Screen Quest Fluo-8 Calcium Assay kit; AAT Bioquest, Sunnyvale, CA, USA) were added, and cells were incubated for 1 h in a 5% CO_2_ incubator. An IX73 fluorescence microscope (Olympus) fitted with an IX3-FGFPXL mirror (Olympus) and an 808-nm NIR laser beam from a CW diode laser (133 mW, or ∼68 μW μm^−2^; Sigma Koki) were used to irradiate cells at 20 °C, with × 20 magnification and 0.75 aperture (UPLSAPO20X, Olympus). Images were recorded on an EM-CCD camera (DP80) before and during irradiation. The green fluorescence of Fluo-8 was monitored in real-time using GFP channel. The fluorescence intensities were analysed using ImageJ. Cell viability was measured in real time by propidium iodide staining (Dojindo, Kumamoto, Japan), using the same microscope system except that an IX2-FGWXL (Olympus) mirror was used.

### PA imaging

A commercial Endra Nexus128 PA tomography system (Endra Inc., Ann Arbor, MI, USA) was also used in this study. The system houses a tunable nanosecond pulsed laser (7 ns pulses, 20 Hz pulse repetition frequency, 9 mJ pulse^−1^ on the animal surface, wavelength range (680−950 nm), 128 unfocused ultrasound transducers (with 5 MHz centre frequency and 3 mm diameter) arranged in a hemispherical bowl filled with water, animal tray on top of the bowl, data acquisition/reconstruction console, servo motors for three-dimensional rotation of the bowl and a temperature monitor of the water bath. To obtain the PA imaging of nanoparticle solutions, nanoparticles were suspended in Matrigel (BD Bioscience, Franklin Lakes, NJ, USA) to different concentrations. The final concentration of Matrigel was 50% vol/vol for all solutions. Matrigel-nanoparticle mixtures (30 μl) were injected subcutaneously on the dorsal aspects of female nude mice to form inclusions of nanoparticles, and PA imaging was performed.

For targeting cancer imaging, Anti-EGFR-Biotin-Avidin-DSPE-PEG_2000_-Amine-DC(8,9)PC-LM (LM concentration=100 μg ml^−1^, 50 μl), or Avidin-DSPE-PEG_2000_-Amine-DC(8,9)PC-LM (LM concentration=100 μg ml^−1^, 50 μl) was injected into tumours (average volume of tumour is about 1,000 mm^3^) in seven-week-old female nude mice (Average weight=19 g, *N*=2; BALB/cSlc-nu/nu, Japan SLC). The PA and US imaging of injected LM nanocapsules in tumour was performed by Primetech, Co. (Tokyo, Japan) using the PA and US instruments (Vevo LAZR system with LZ-550 probe; Fujifilm-VisualSonics, Inc., Tronto, Canada) under anaesthesia.

### Data availability

The data that support the findings of this study are available from the corresponding author on request.

## Additional information

**How to cite this article:** Chechetka, S. A. *et al*. Light-driven liquid metal nanotransformers for biomedical theranostics. *Nat. Commun.* **8,** 15432 doi: 10.1038/ncomms15432 (2017).

**Publisher's note:** Springer Nature remains neutral with regard to jurisdictional claims in published maps and institutional affiliations.

## Supplementary Material

Supplementary Information Supplementary Figures and Supplementary References.

Supplementary Movie 1 Light-driven transformation of LM nanocapsules *in vitro*.

Supplementary Movie 2 Thermal expansion of a LM droplet in air by laser irradiation.

Supplementary Movie 3 Laser-induced morphological changing of a LM droplet in NaOH solution.

Supplementary Movie 4 Photo-promoted morphological changing of LM aqueous suspension.

Supplementary Movie 5 Thermal expansion of LM droplet by heating. The movie is edited at 10 times speed compared to the original.

Supplementary Movie 6 Laser-induced transformation of LM nanocapsules in microfluidic device.

Supplementary Movie 7 Controlling temperature of microchannel by laser-induced LM nanocapsules.

Supplementary Movie 8 3D-X-ray mapping of laser-induced LM nanocapsules in organs.

Supplementary Movie 9 3D-X-ray mapping of laser-induced LM nanocapsules in a living mouse.

Supplementary Movie 10 Real-time elimination of HeLa cells by photo-induced LM nanocapsules.

Supplementary Movie 11 Spatiotemporal stimulation of ND7/23 cells by photo-induced LM nanocapsules.

Supplementary Movie 12 Spatiotemporal stimulation of HeLa cells by photo-induced LM nanocapsules.

Supplementary Movie 13 Stimulation of ND7/23 cells by multiple laser irradiation events (ON-OFF).
