# Supplementary material for: Light-driven liquid metal nanotransformers for biomedical theranostics
Source: Nat Commun. 2017 May 31;8:15432. doi: 10.1038/ncomms15432 (PMC5460022; doi:10.1038/ncomms15432)
Supplement: Supplementary Information — Supplementary Figures and Supplementary References. [file ncomms15432-s1.pdf]

Supplementary Figures

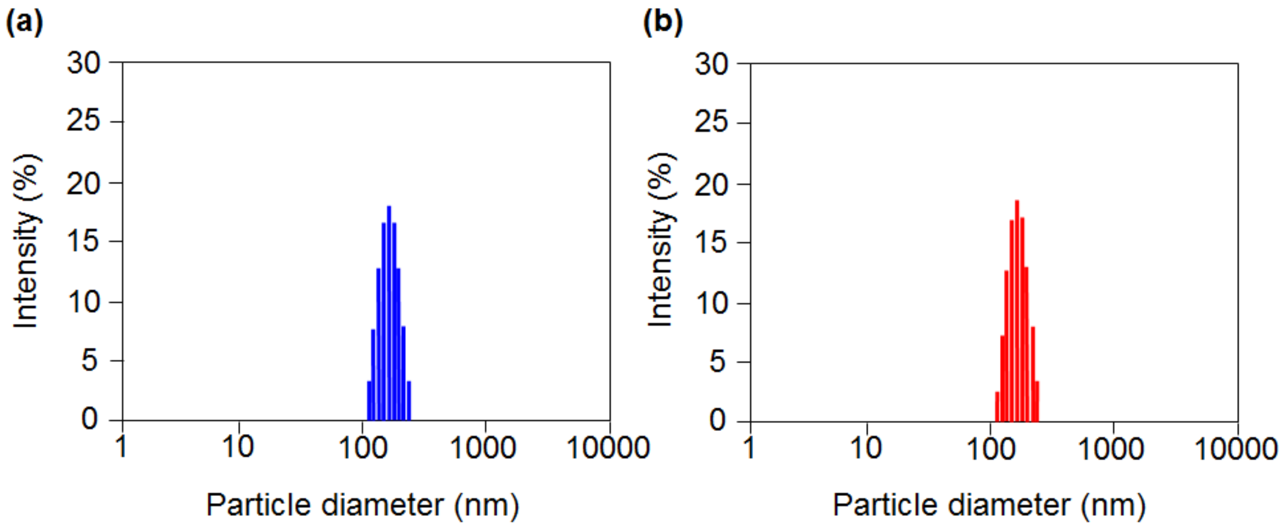

Supplementary Figur e 1. DLS analyses of DSPE–PEG_{2000}–Amine–DC(8,9)PC–LM (a) immediately after its preparation and (b) after 3 days.

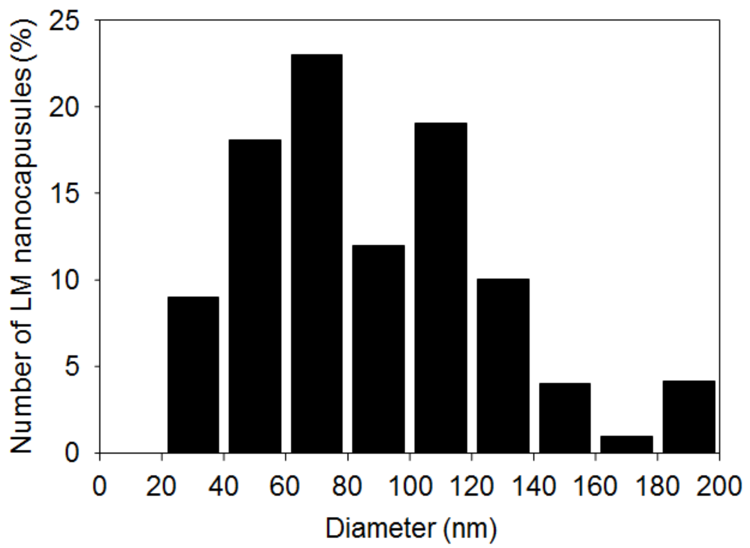

Supplementary Figure 2. Particle size distribution of LM nanocapsules (N = 100) from TEM observation.

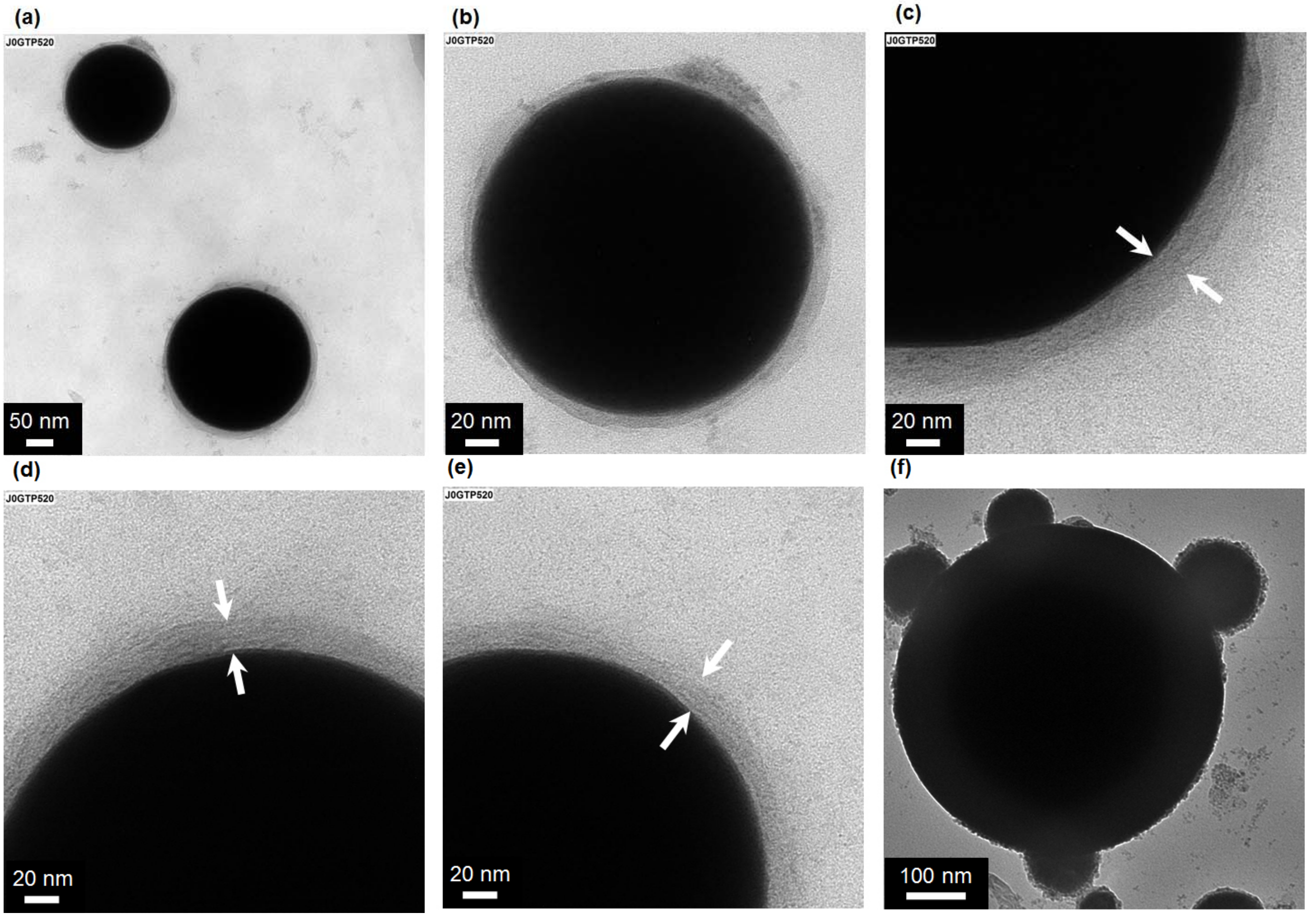

Supplementary Fig ure 3 . TEM images of (a–e) DSPE–PEG$_{2000}$–Amine–DC(8,9)PC–LM and (f) DSPE–PEG$_{2000}$–Amine–LM. White arrows represent polymeric shell structure of UV-cured DSPE–PEG$_{2000}$–Amine and DC(8,9)PC molecules.

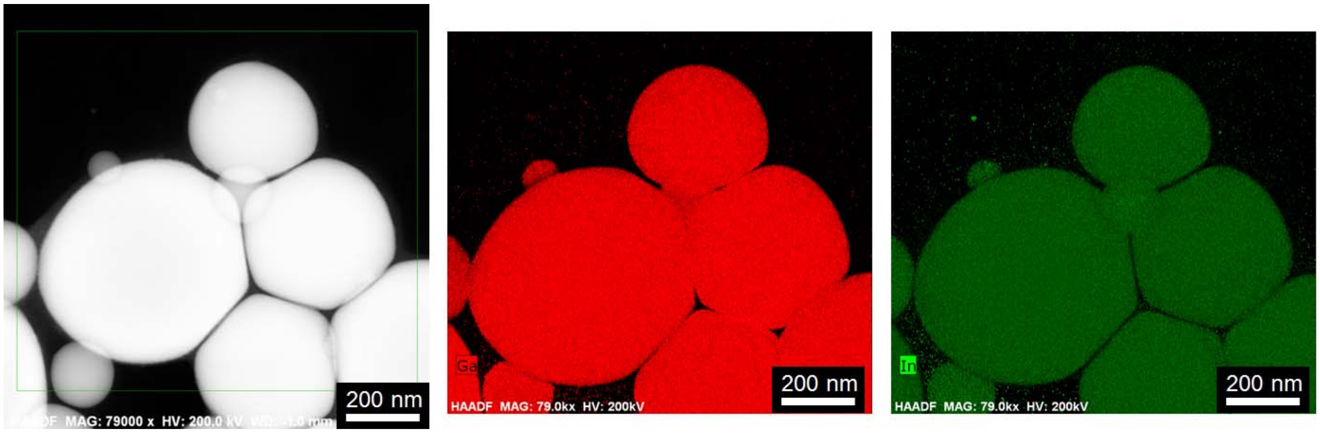

Supplementary Figure 4. STEM/EDS mapping of DSPE–PEG$_{2000}$–Amine–DC(8,9)PC–LM nanocapsules.

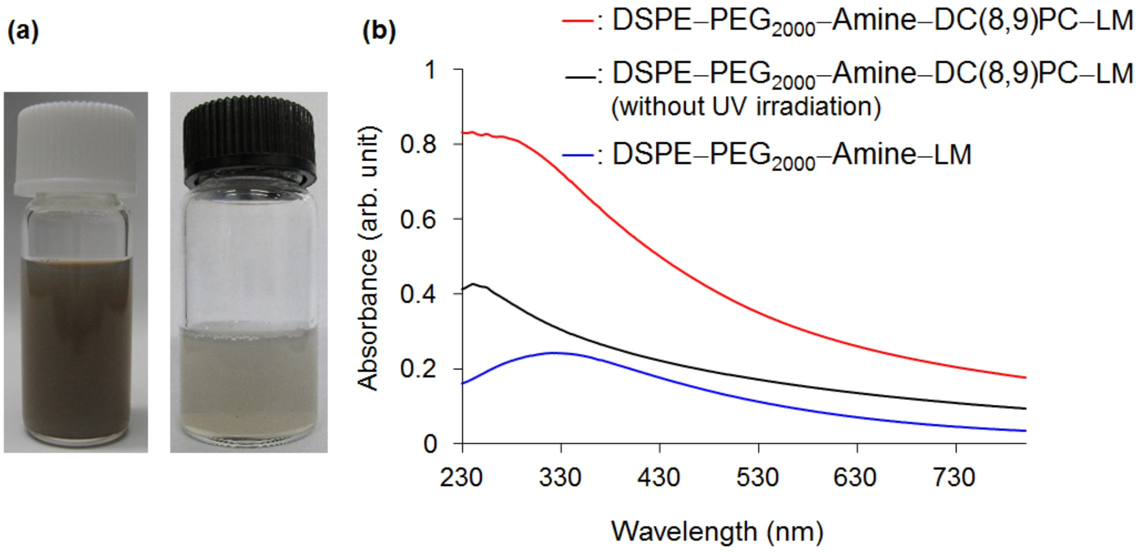

Supplementary Figure 5. Water dispersibility of LM nanocapsules. (a) Aqueous suspensions of (left) DSPE–PEG$_{2000}$–Amine–DC(8,9)PC–LM and (right) DSPE–PEG$_{2000}$–Amine–LM. (b) UV-Vis-NIR absorbance of LM nanoconjugates after centrifugation at 1,000 rpm for 10 min at 20 °C.

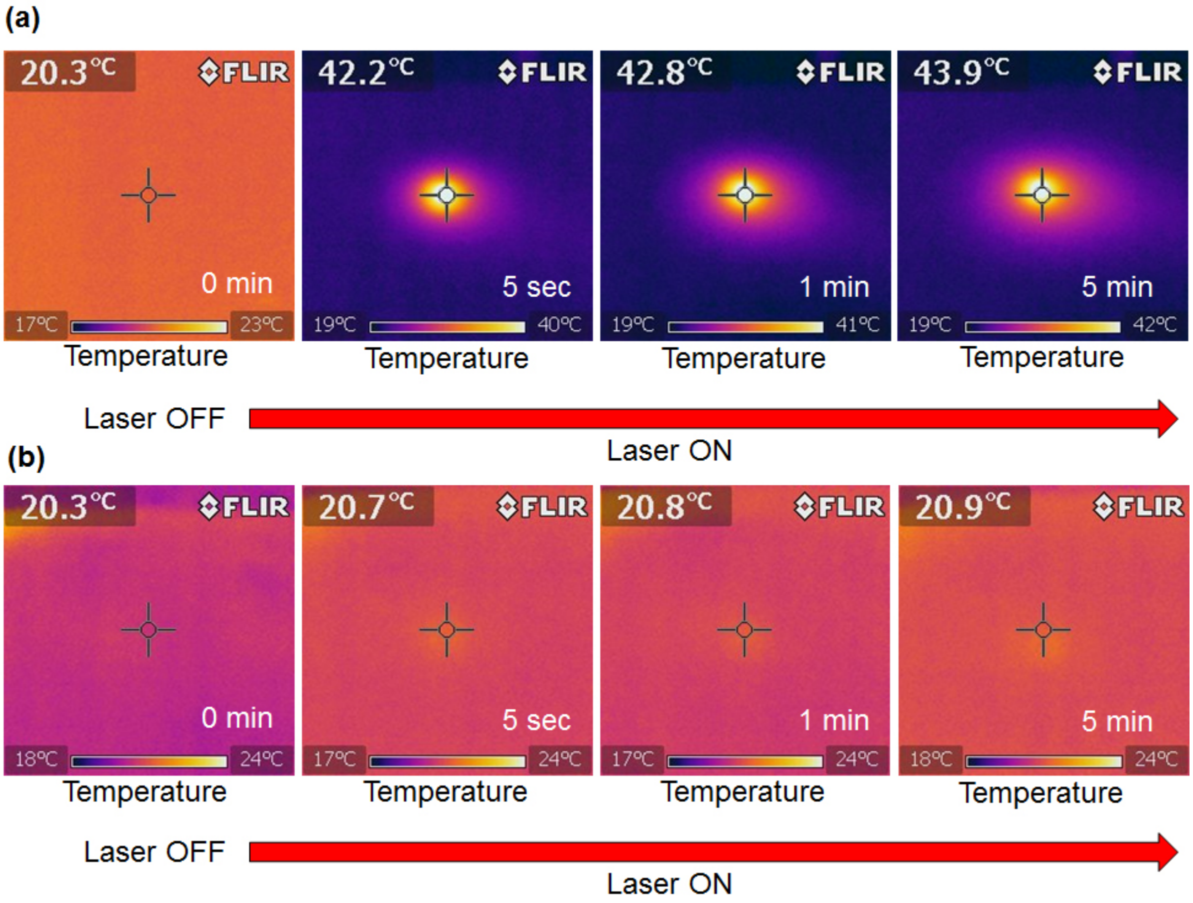

Supplementary Figure 6. Thermographic images on the surface of (a) a representative LM droplet (1 mg) and (b) filter paper, using a 785-nm NIR laser at 1 W (~80 mW mm^{-2}) for 5 min.

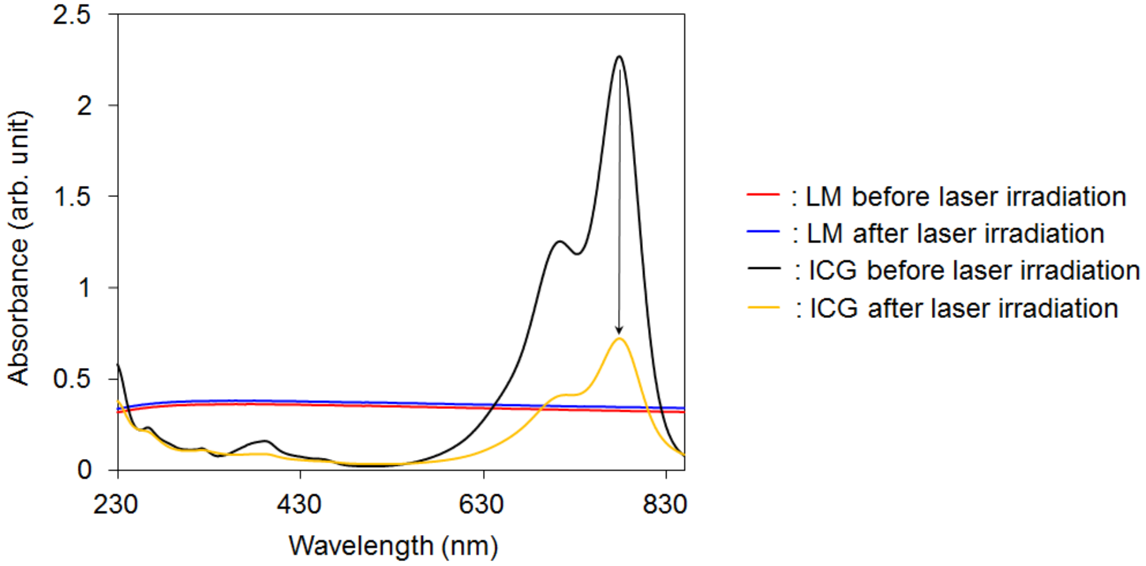

Supplementary Figure 7. Photothermal stability of LM after 785-nm NIR laser irradiation at 1 W (~80 mW mm^{-2}) for 1 hour. LM was dispersed in water by DSPE–PEG_{2000}–Amine.

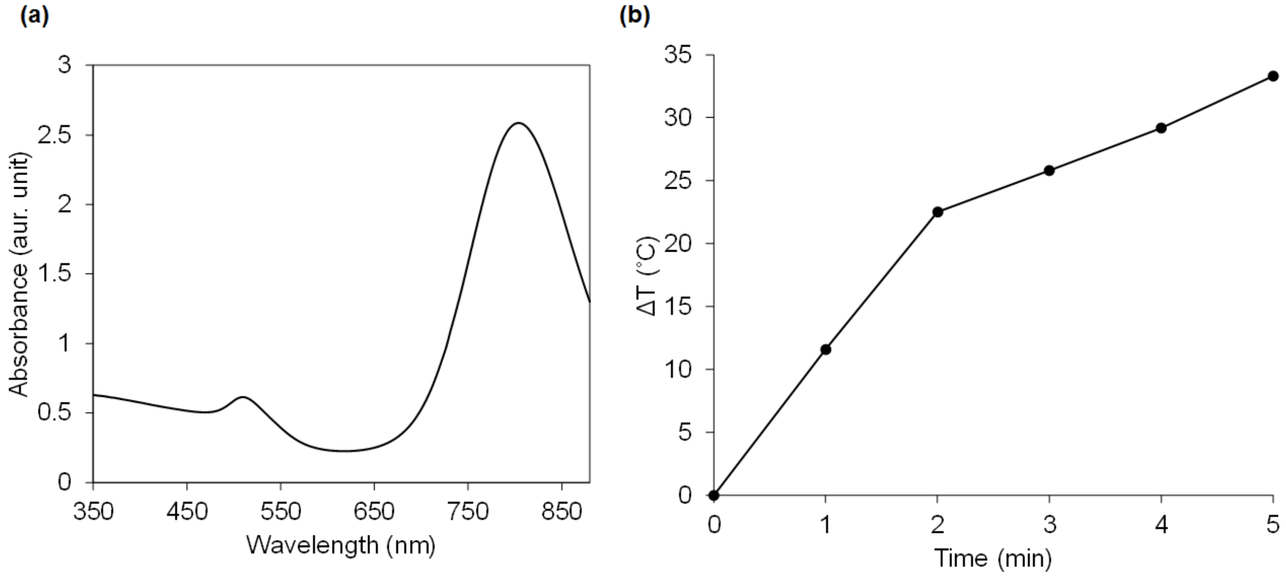

Supplementary Figure 8. (a) Optical absorbance and (b) photothermal property of Au-NR1 solution after 785-nm NIR laser irradiation at 1 W (~80 mW mm^{-2}) for 5 min. Concentration of Au-NR1 = 100 µg ml^{-1}.

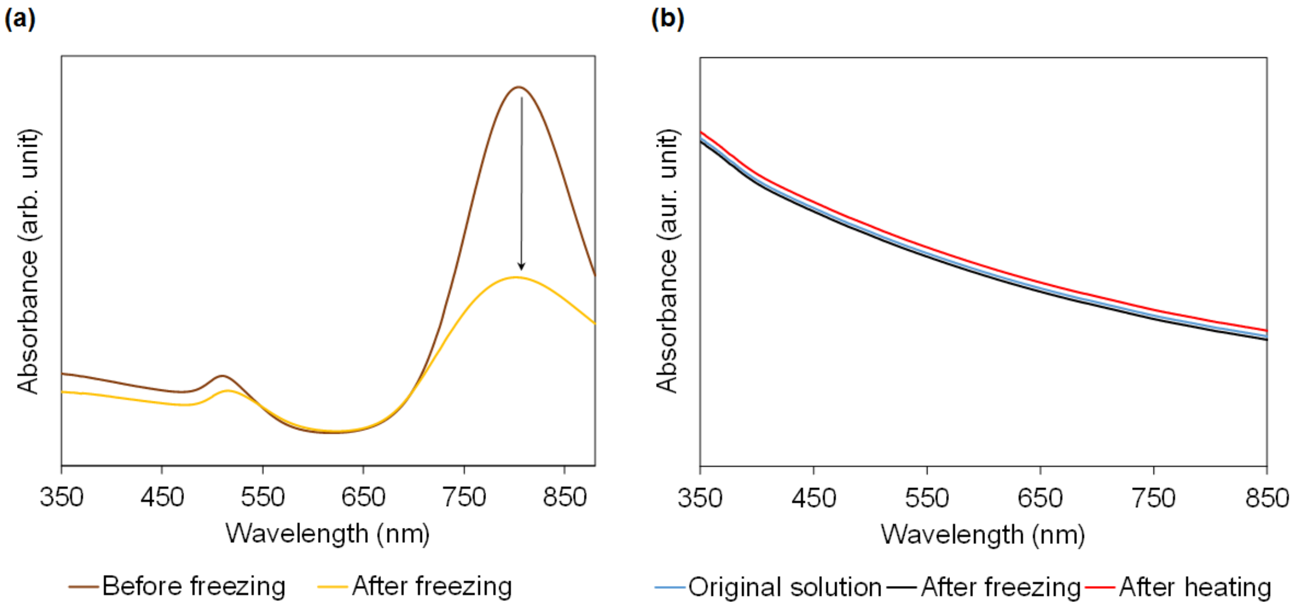

Supplementary Figure 9. (a) Optical absorbance of Au-NR1 before (brown line) and after freezing (yellow line). (b) Heating and freezing resistances of LM nanocapsules. Concetrions of LM and Au-NR were adjusted at 500 μg ml^{-1}. Temperature of heating and freezing were 80 and -80˚C, respectively.

It is well known that Au-NRs are very unstable against freezing and heating due to the shape deformations (degradation).[1,2] Indeed, optical absorbance of Au-NR1 was obviously decreased when it was frozen in an aqueous solution (-80˚C) for 1 hour because of coalescent reshaping of Au-NRs (Supplementary Figure 9a).[1,2] These molecular degradations could be caused to reduce the photothermal conversion efficiency of Au-NRs.

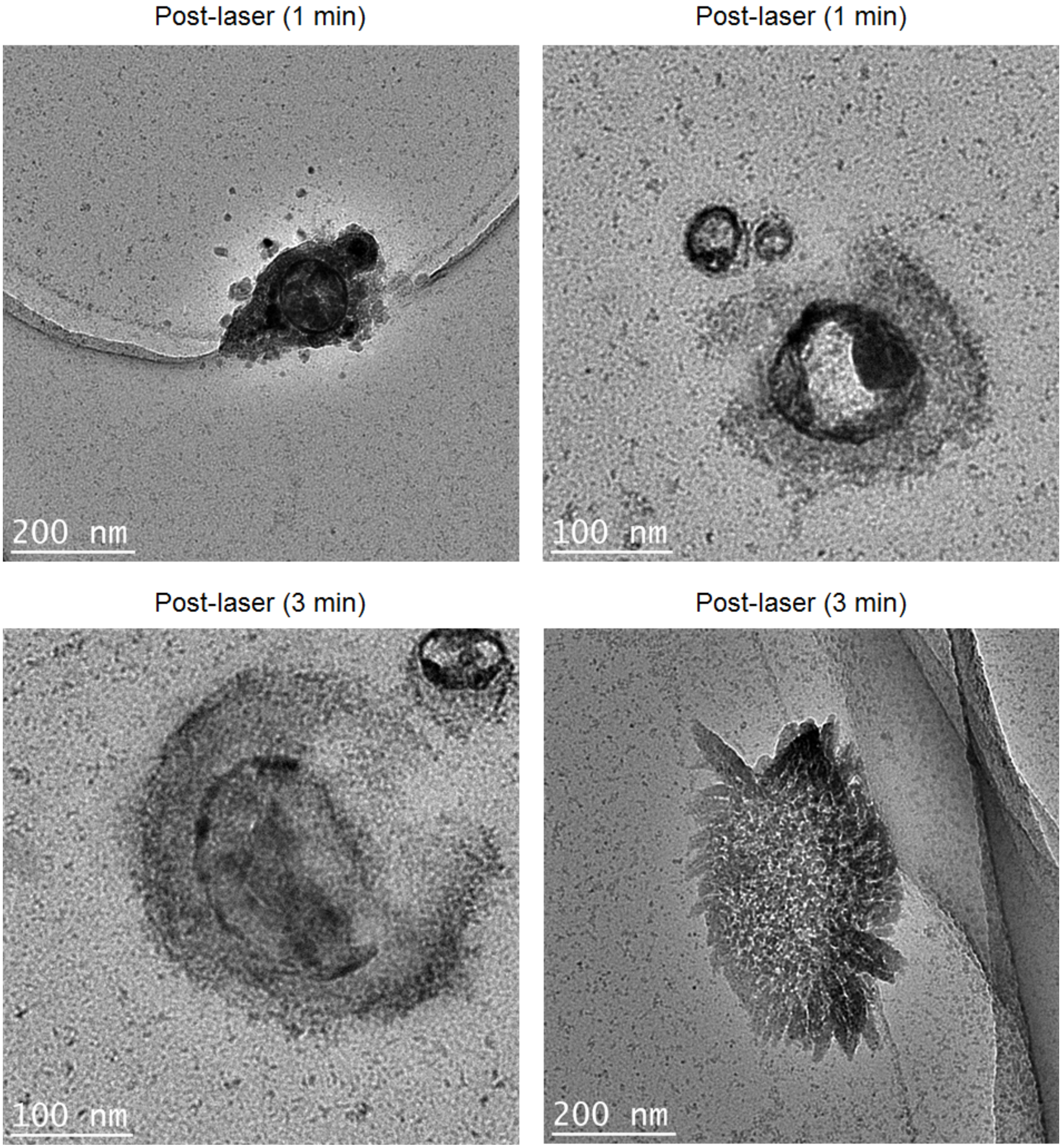

Supplementary Figure 10. TEM images of DSPE–PEG_{2000}–Amine–DC(8,9)PC–LM after laser irradiation for 1 or 3 min (785 nm, 1 W, ~80 mW mm^{-2}).

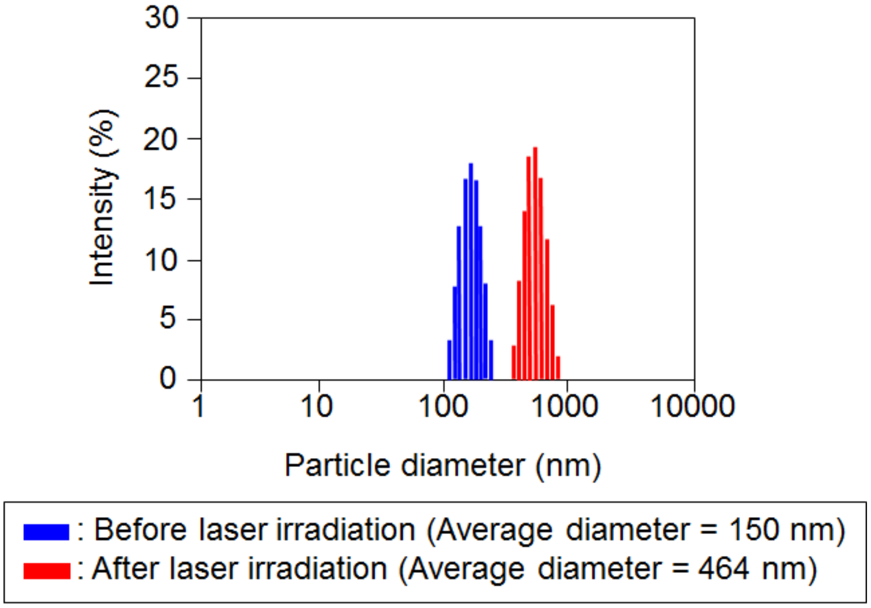

Supplementary Figure 11. DLS analyses of DSPE–PEG_{2000}–Amine–DC(8,9)PC–LM before and laser irradiation for 1 h (785 nm, 1 W, ~80 mW mm^{-2}).

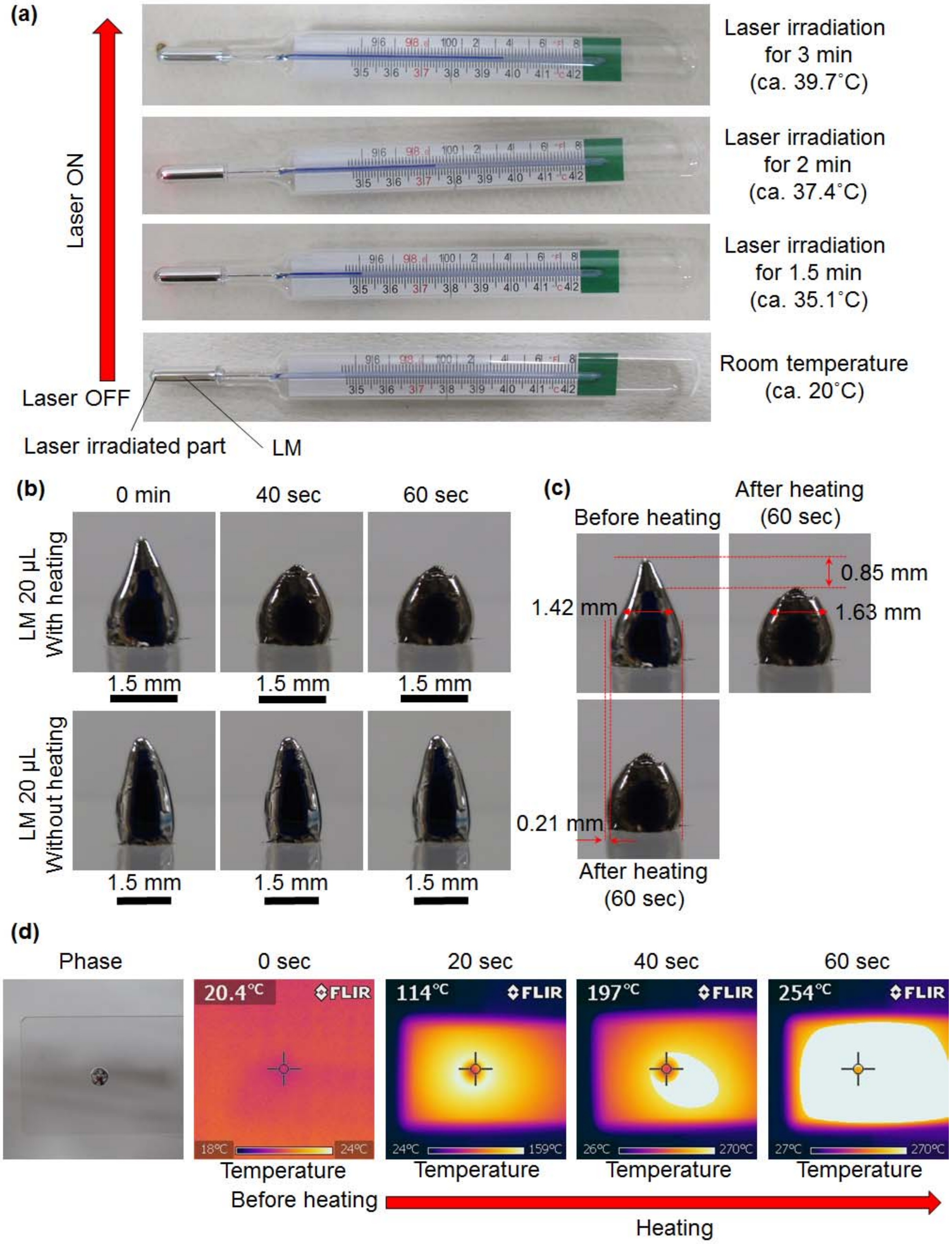

Supplementary Fig ure 12. Thermal expansion of LMs. (a) Laser-induced temperature changing and thermal expansion of a Galinstan thermometer. (b and c) Thermal expansion and shape changing of an EGaIn LM droplet by heating. (d) Thermographic images on the surface of a LM droplet during heating.

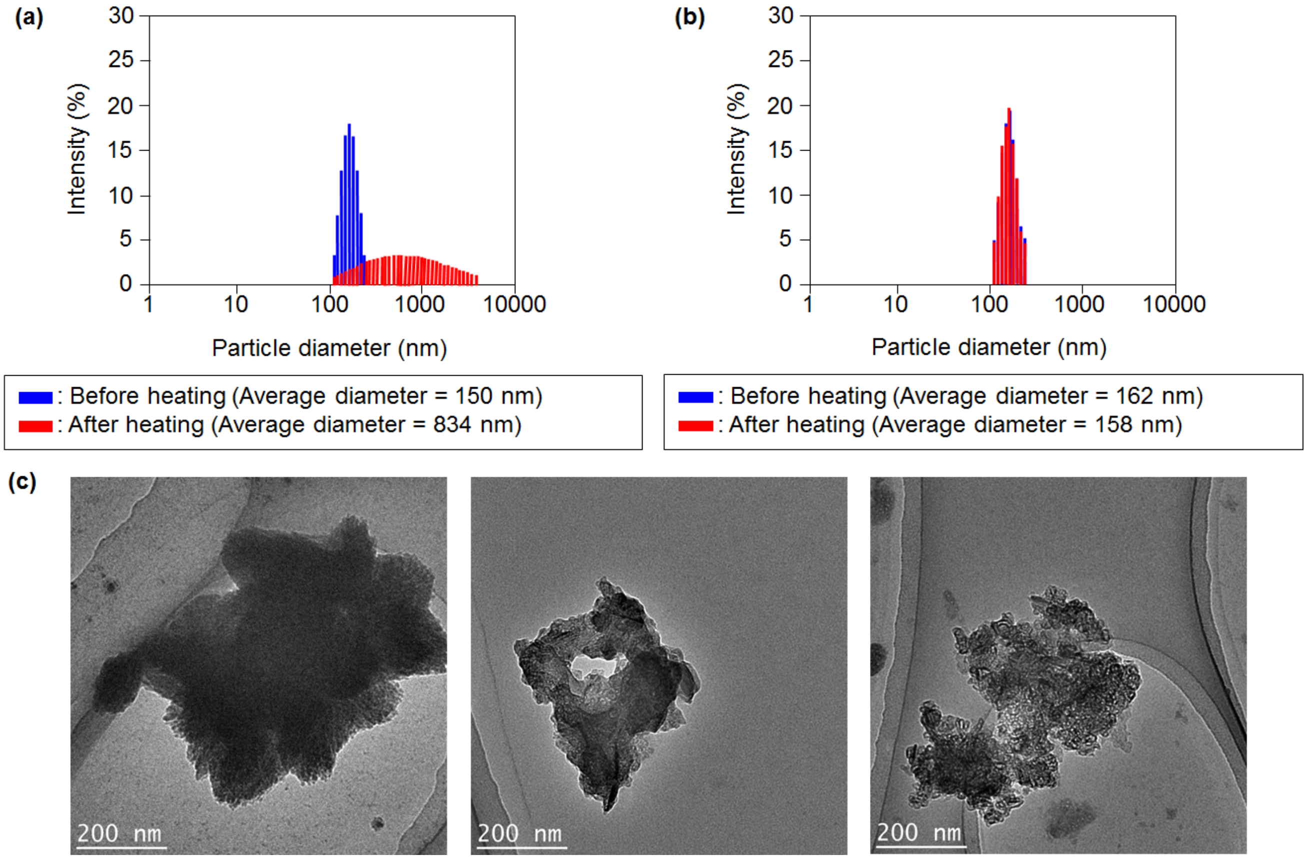

Supplementary Figure 13. DLS analyses of (a) LM nanocapsule and (b) DSPE-PEG$_{2000}$-Amine and DC(8,9)PC nanocapsule without LM before and after heating at 130˚C for 30 min. (c) TEM images of LM nanocapsules after heating at 130˚C for 30 min.

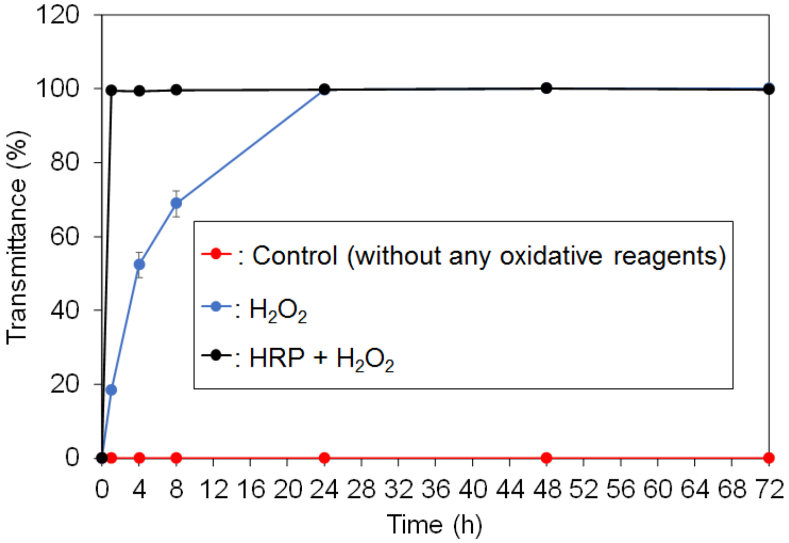

Supplementary Figure 14. Degradation of LM nanocapsules by H_2O_2- and enzymatic-oxidative treatments. Error bars represent standard deviations of three separate measurements.

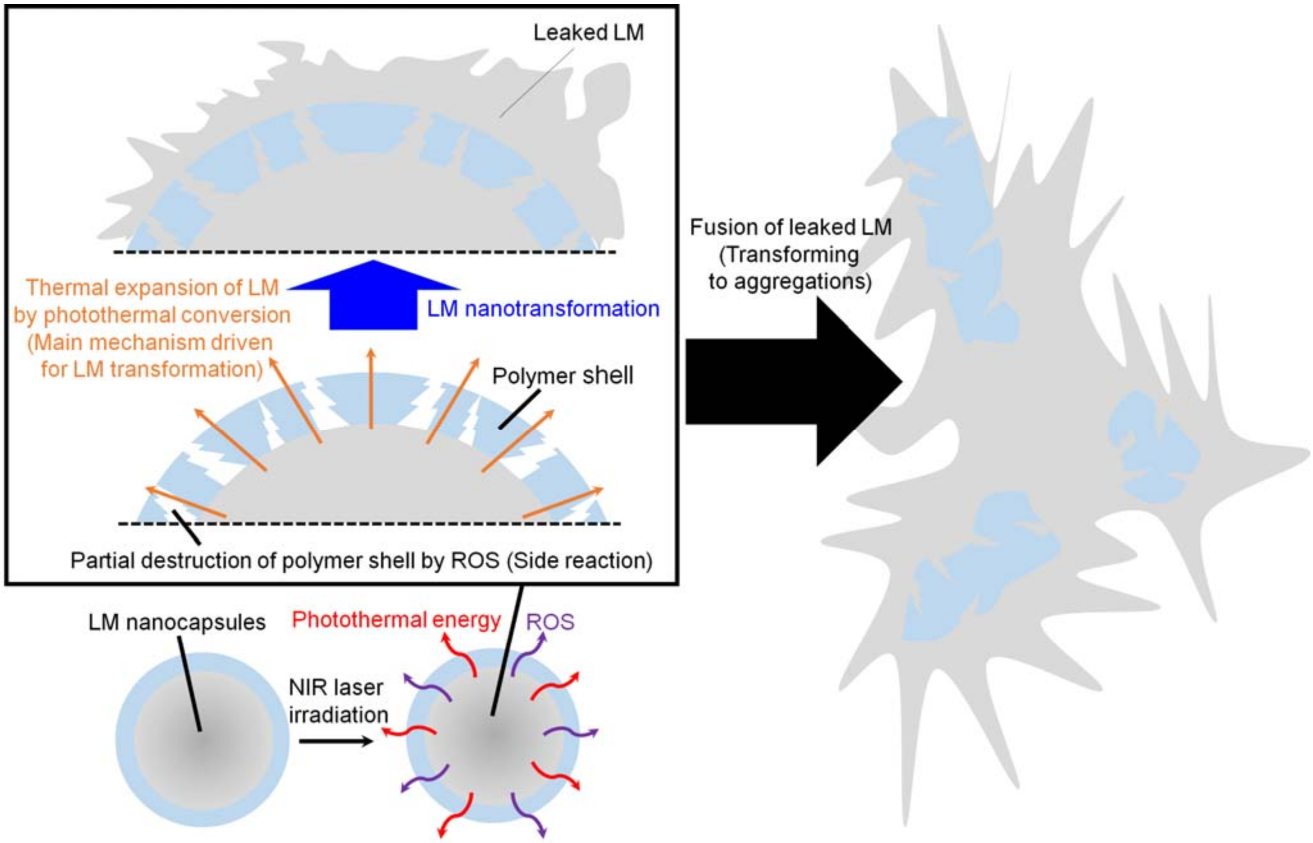

Supplementary Figu re 15 . Proposed mechanism for laser-induced disintegration and transformation of LM nanocapsules.

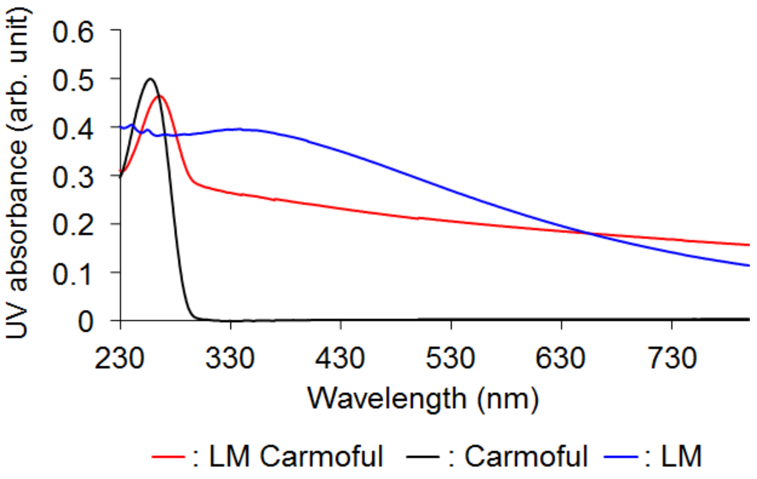

Supplementary Figur e 16. UV–Vis–NIR absorbance of DSPE–PEG_{2000}–Amine–DC(8,9)PC–LM encapsulating carmofur in water (red line; LM concentration: 270 μg ml^{-1}), DSPE–PEG_{2000}–Amine–DC(8,9)PC–LM in water (blue line; LM concentration: 270 μg ml^{-1}), and carmofur in ethanol (black line; carmofur concentration: 267 μg ml^{-1}).

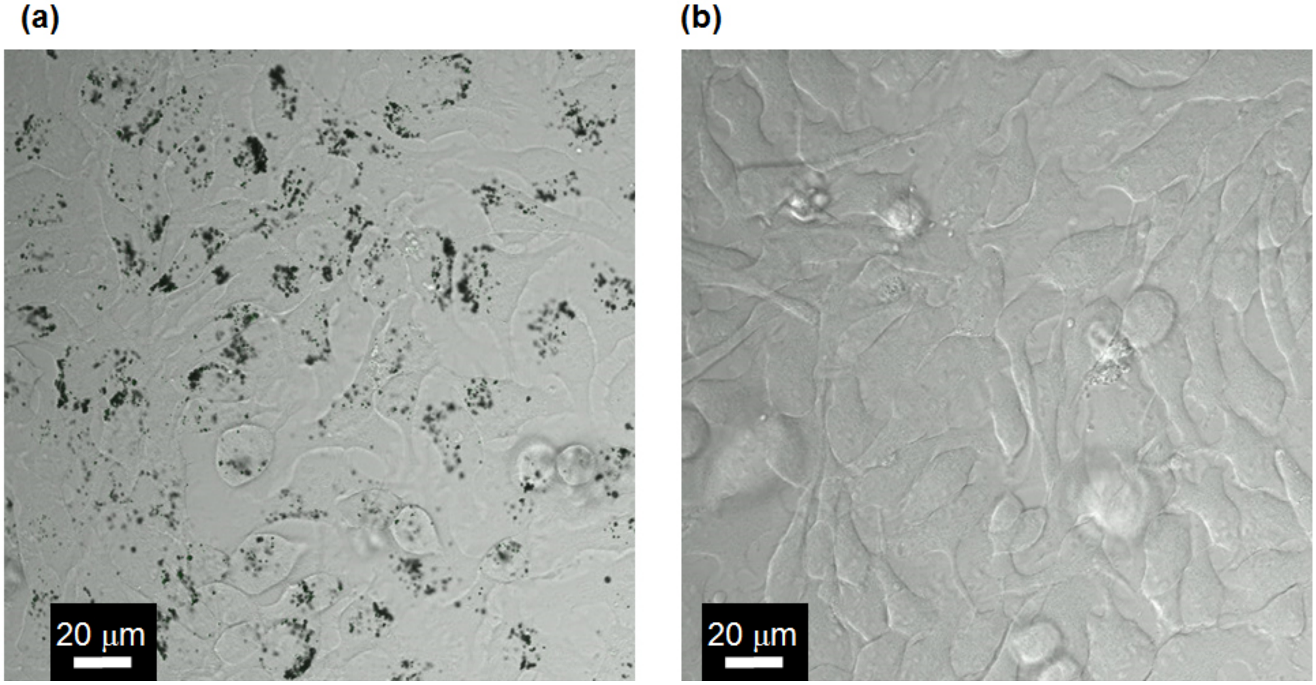

Supplementary Figur e 17. Confocal microscopic differential-interference-contrast (DIC) images of HeLa cells incubated for 4 h with (a) DSPE–PEG_{2000}–Amine–DC(8,9)PC–LM (LM concentration: 100 μg/mL) and (b) control without LM nanocapsules. Objective, 40×.

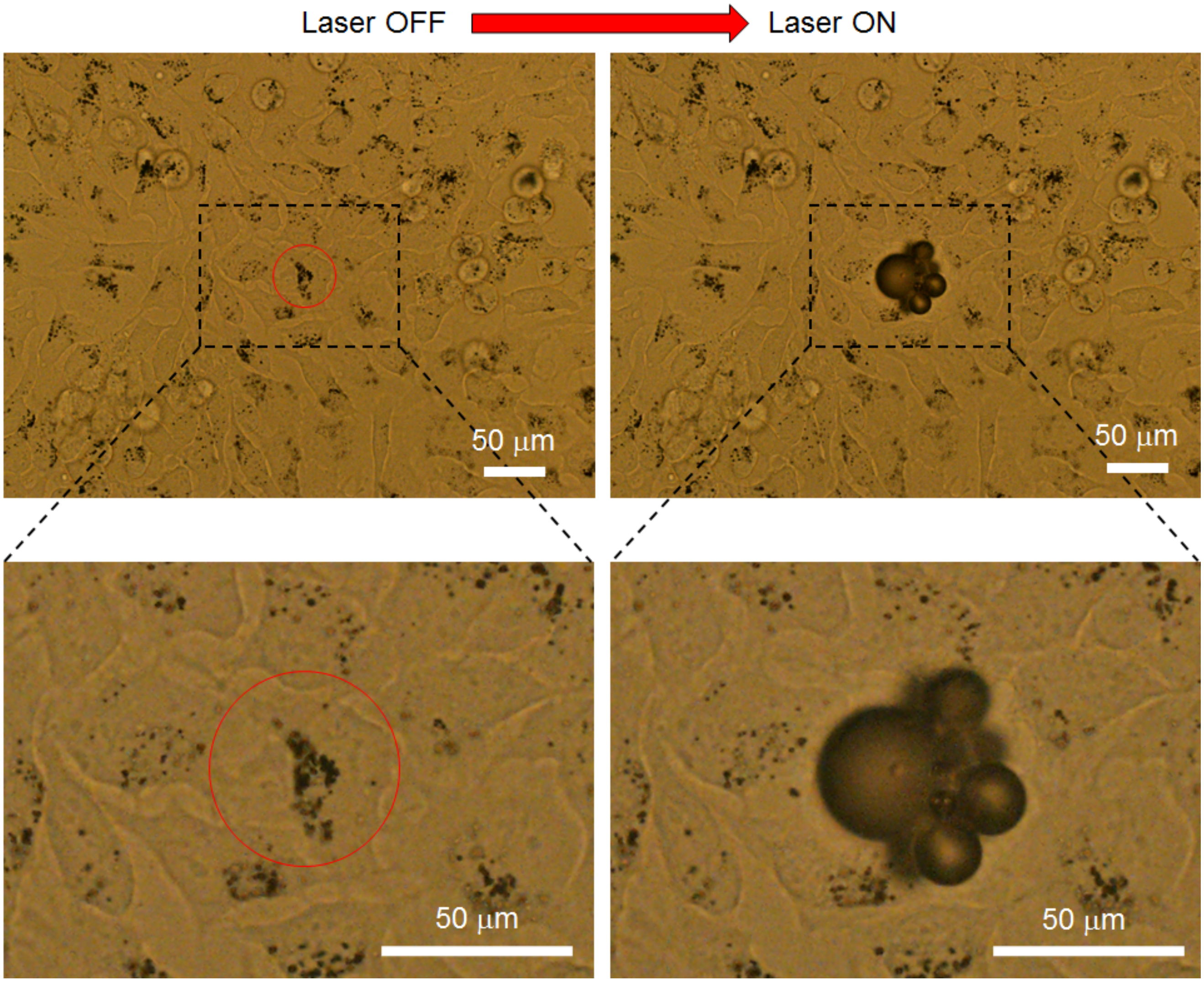

Supplementary Figure 18. Real-time elimination of HeLa cells caused by laser-induced LM nanocapsules before and after laser irradiation (808 nm, 564 mW, ~287 μW μm^{-2}). The red circle shows laser irradiation position and area.

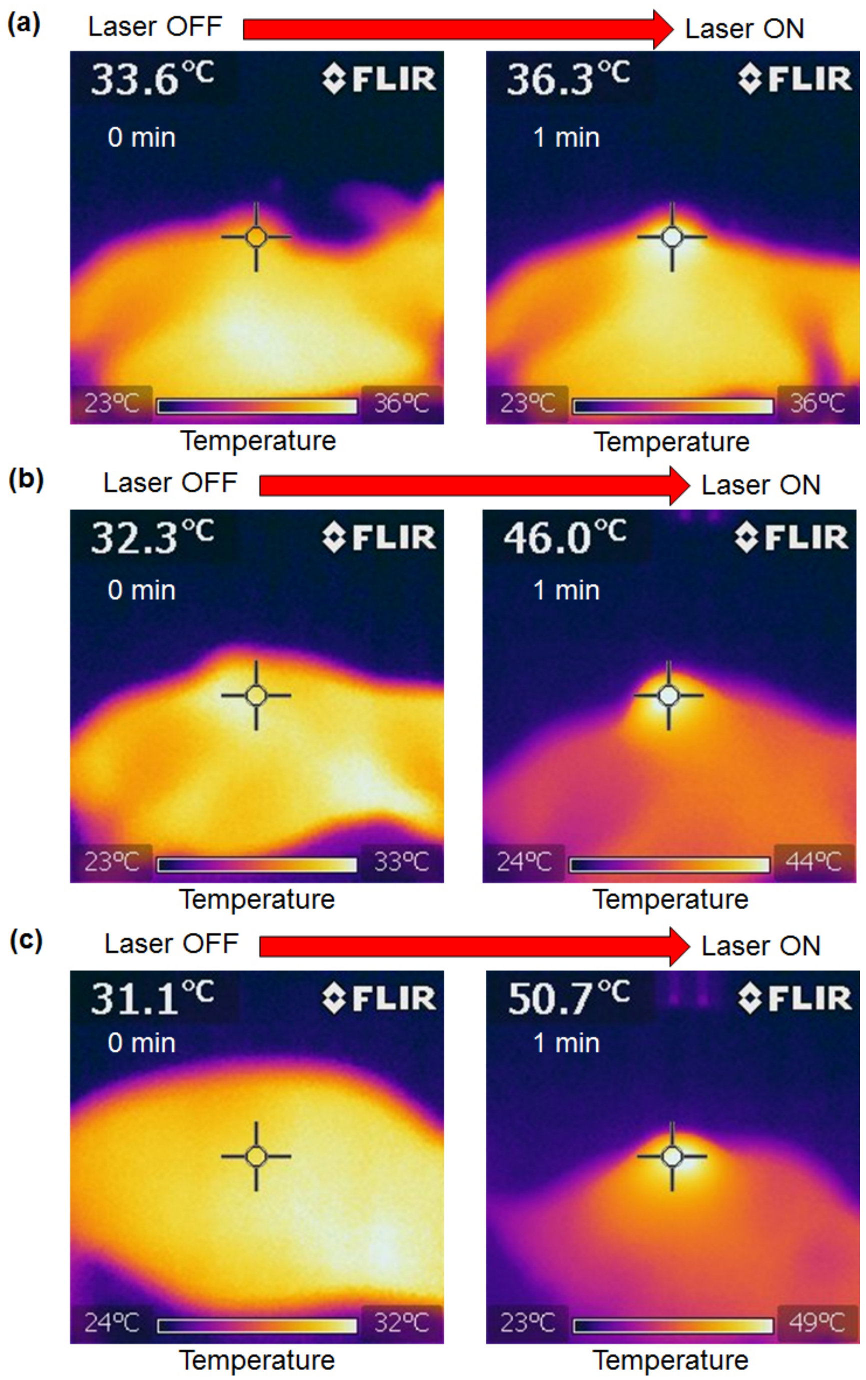

Supplementary Fig ure 19 . Surface temperature of tumors treated with injections of (a) HEPES, (b) Avidin–DSPE–PEG$_{2000}$–Amine–DC(8,9)PC–LM, and (c) Anti-EGFR-Biotin–Avidin–DSPE–PEG$_{2000}$–Amine–DC(8,9)PC–LM after laser irradiatin for 1 min.

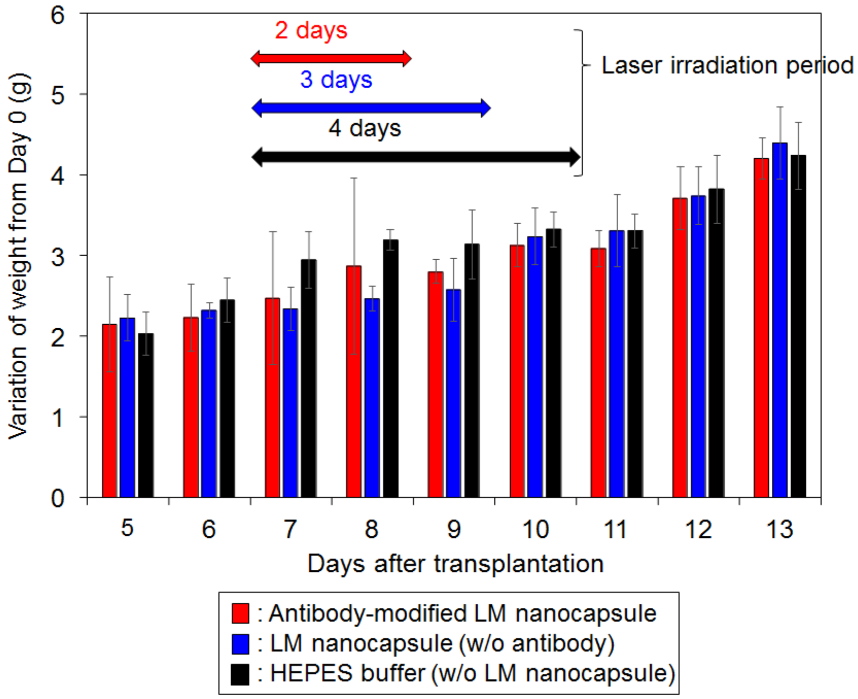

Supplementary Fig ure 20. Relative body weight of mice in the cancer phototherapy testing period. Error bars represent standard deviations of measurements from nine mice.

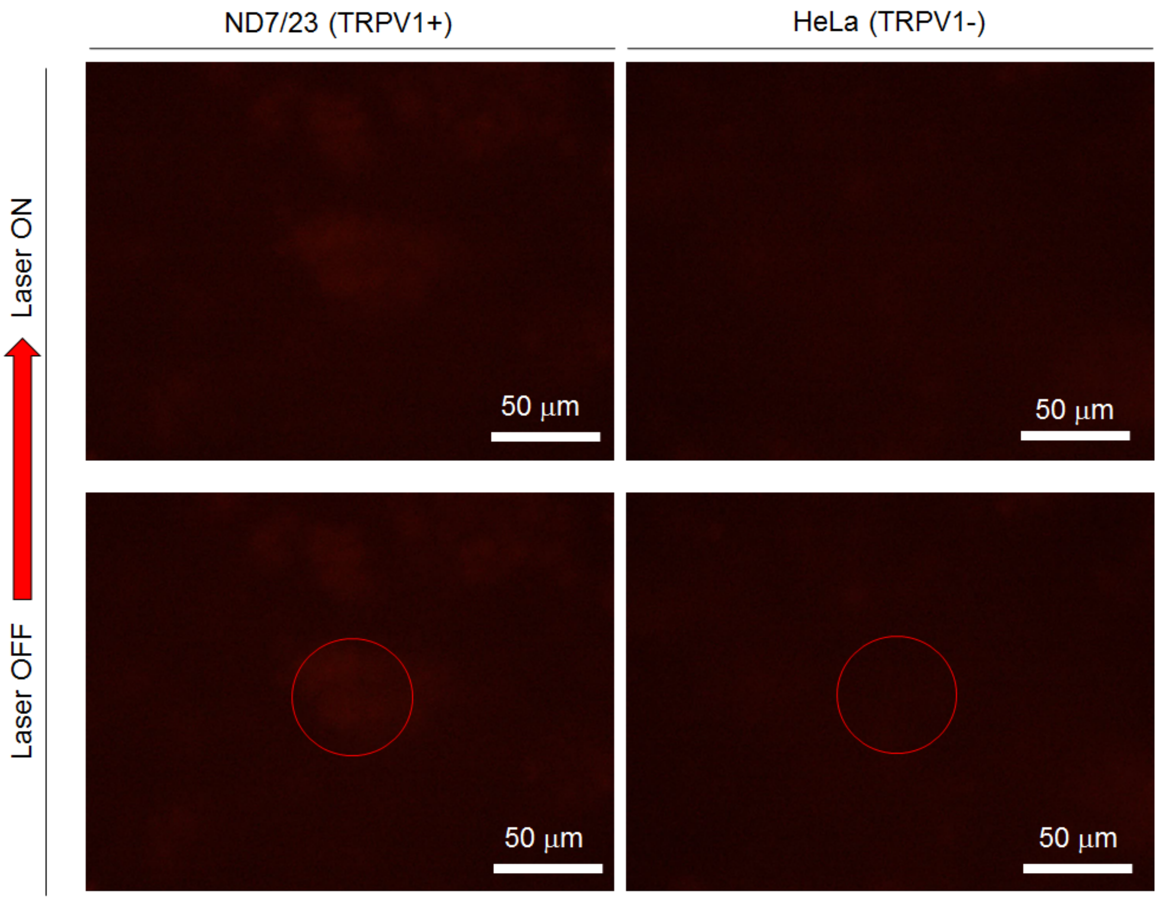

Supplementary Figure 21. Propidium iodide staining for dead cells. Magnification, 20×; (808 nm; laser 133 mW; 68 $\mu W\ \mu m^{-2}$). Irradiated areas are marked in red.

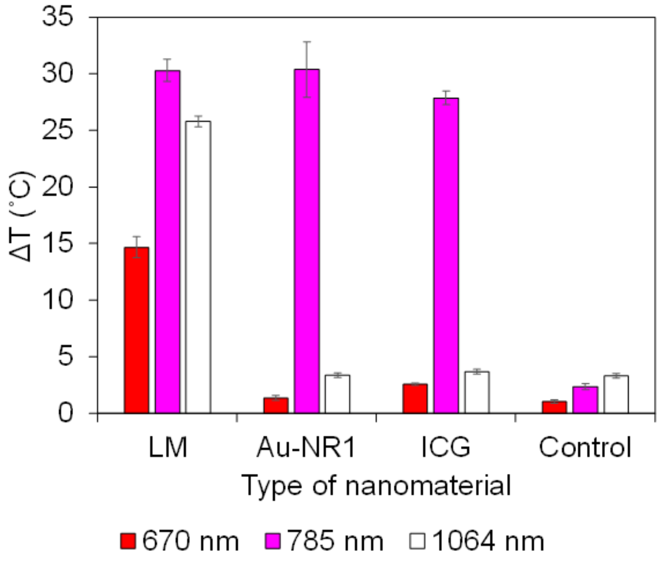

Supplementary Figure 22. Photothermal conversion of LM nanocapsules by various wavelengths laser irradiation. Data represent the mean of three determinations; error bars show the SD.

Photothermal conversion of LM nanocapsules was able to be conducted over a wide range of irradiation wavelengths (670, 785, and 1064 nm) (Supplementary Figure 22). Meanwhile, conventional NIR reagents (Au-NR1 and ICG) could be available by exciting with only monochromic light source at 785 nm (Supplementary Figure 22). Wide-ranging analyses generally require the use of a wide range of wavelengths of excitation laser. Thus, the photothermal property of LM nanocapsules would be induced and controlled at the desired wavelengths by exploiting suitable lasers for these wide-ranging analytical methods. More importantly, the utilization of second NIR optical window (NIR-II, 1000-1700 nm, also be divided into NIR-IIa and NIR-IIb separated by 1400 nm[3,4]) light leads to promising applications of LM nanocapsules treating deep-tissue-buried diseases or supplying energy to subcutaneous implantable bioelectronic devices.[5] However, only a few examples of NIR-II materials have been demonstrated, involving several photothermal compounds, such as Au nanoparticles[6–8] and organic dye molecules[3,4].

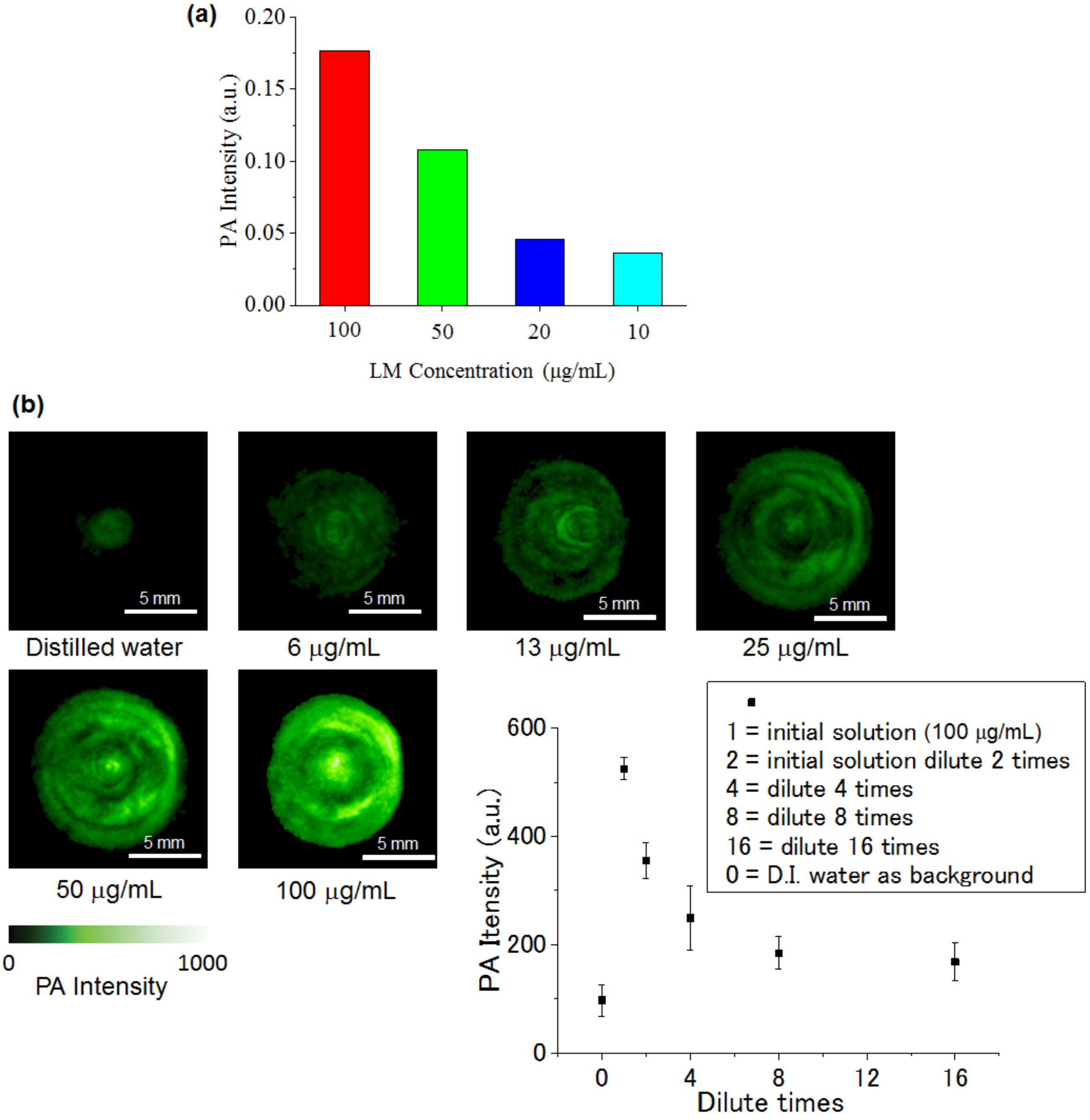

Supplementary Figure 23. (a) PA intensity of various LM nanocapsule concentrations at an excitation wavelength of 680 nm. (b) *Ex vivo* PA imaging of phantom samples. Error bars represent standard deviations of three separate measurements. Excitation wavelength of laser is 680 nm.

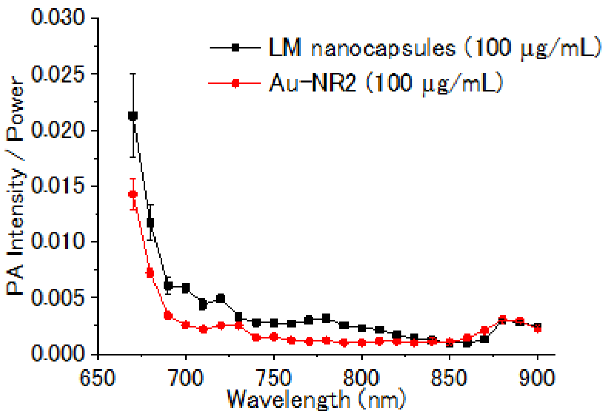

Supplementary Figur e 24. PA intensity of LM nanocapsule and Au-NR2 at various excitation wavelength. Data represent the mean of three determinations; error bars show the SD.

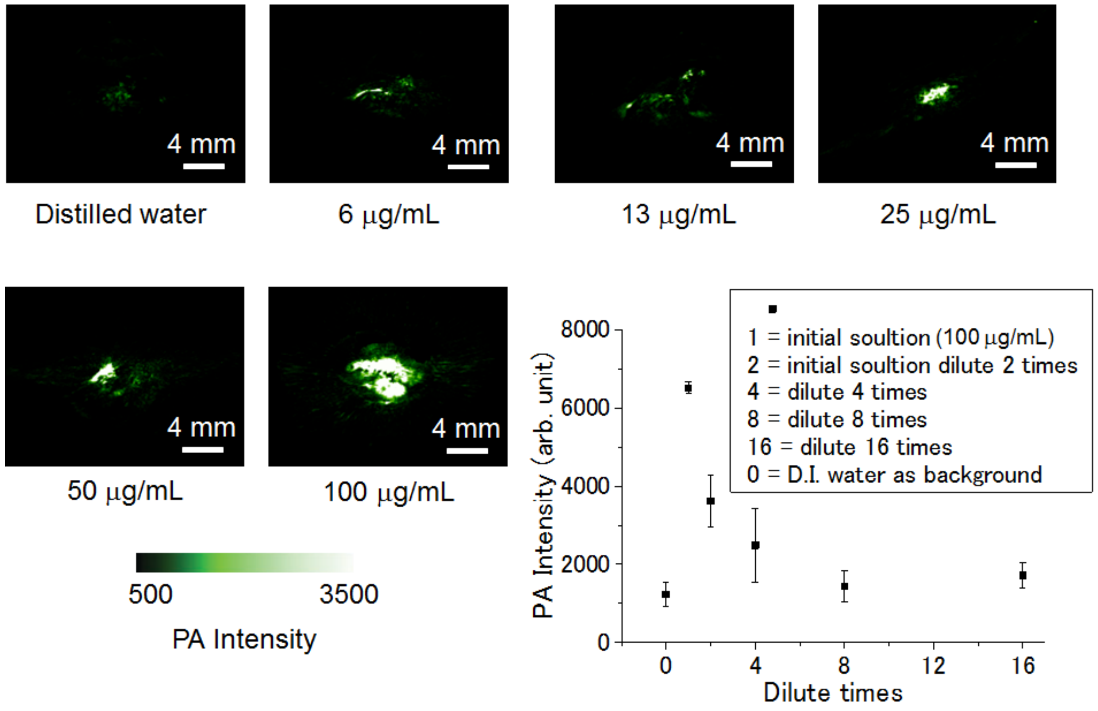

Supplementary Figure 25. Effect of various concentrations of LM nanocapsules on the PA signal *in vivo*. Error bars represent standard deviations of measurements from three mice. Excitation wavelength of laser is 680 nm.

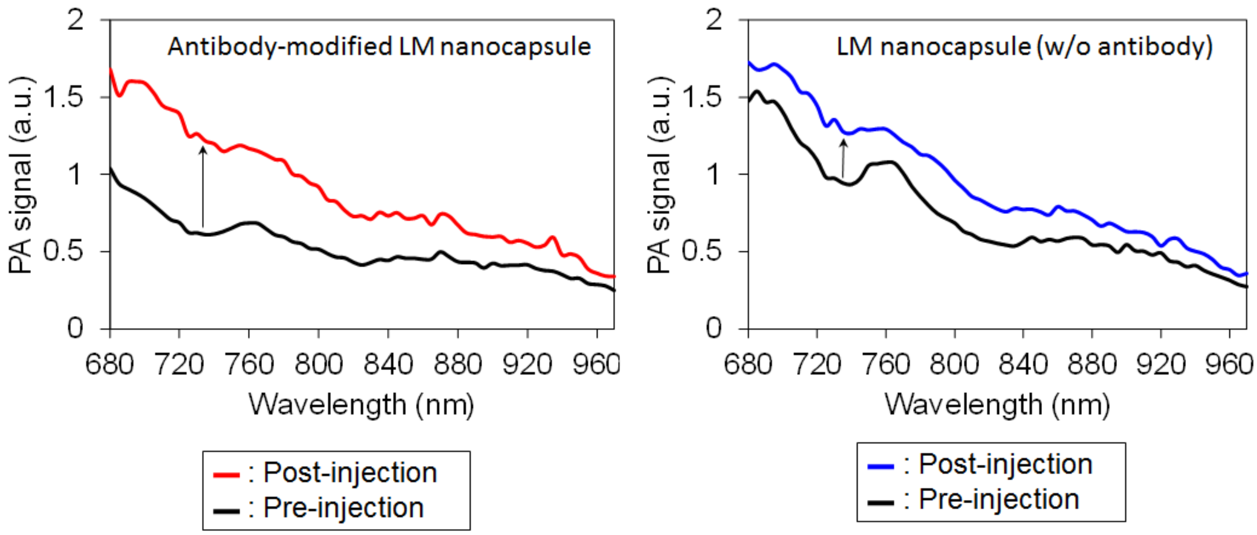

Supplementary Figure 26. PA signal in tumors treated with antibody-functionalised LM and plain LM nanocapsules before and after injections.

Supplementary References

1. Albert, G. C., Roumeliotis, M. & Carson, J. J. L. The effect of temperature and freeze–thaw processes on gold nanorods. *Nanotechnology* **20**, 505502 (2009).
2. Zou, R., Zhang, Q., Zhao, Q., Peng, F., Wang, H., Yu, H. & Yang, J. Thermal stability of gold nanorods in an aqueous solution. *Colloids Surf A Physicochem Eng Asp.* **372**, 177–181 (2010).
3. Zhang, X.-D., Wang, H., Antaris, A. L., Li, L., Diao, S., Ma, R., Nguyen, A., Hong, G., Ma, Z., Wang, J., Zhu, S., Castellano, J. M., Wyss-Coray, T., Liang, Y., Luo, J. & Dai, H. Traumatic brain injury imaging in the second near-infrared window with a molecular fluorophore. *Adv. Mater.* **28**, 6872–6879 (2016).
4. Diao, S., Blackburn, J. L., Hong, G., Antaris, A. L., Chang, J., Wu, J. Z., Zhang, B., Cheng, K., Kuo, C. J. & Dai, H. Fluorescence imaging in vivo at wavelengths beyond 1500 nm. *Angew. Chem. Int. Ed.* **54**, 14758–14762 (2015).
5. Miyako, E., Hosokawa, C., Kojima, M., Yudasaka, M., Funahashi, R., Oishi, I., Hagihara, Y., Shichiri, M., Takashima, M., Nishio, K. & Yoshida, Y. A photo-thermal-electrical converter based on carbon nanotubes for bioelectronic applications. *Angew. Chem. Int. Ed.* **50**, 12266–12270 (2011).
6. Tsai, M.-F., Chang, S.-H. G., Cheng, F.-Y., Shanmugam, V., Cheng, Y.-S., Su, C.-H. & Yeh, C.-S. Au nanorod design as light-absorber in the first and second biological near-infrared windows for in vivo photothermal therapy. *ACS Nano* **7**, 5330–5342 (2013).
7. Vijayaraghavan, P., Liu, C.-H., Vankayala, R., Chiang, C.-S. & Hwang, K. C. Designing multi-branched gold nanoechinus for NIR light activated dual modal photodynamic and photothermal therapy in the second biological window. *Adv. Mater.* **26**, 6689–6695 (2014).
8. Ding, X., Liow, C. H., Zhang, M., Huang, R., Li, C., Shen, H., Liu, M., Zou, Y., Gao, N., Zhang, Z., Li, Y., Wang, Q., Li, S. & Jiang, J. Surface plasmon resonance enhanced light absorption and photothermal therapy in the second near-infrared window. *J. Am. Chem. Soc.* **136**, 15684–15693 (2014).
